# Loss of *l(3)mbt* leads to acquisition of the ping-pong cycle in *Drosophila* ovarian somatic cells

**DOI:** 10.1101/gad.283929.116

**Published:** 2016-07-15

**Authors:** Tetsutaro Sumiyoshi, Kaoru Sato, Hitomi Yamamoto, Yuka W. Iwasaki, Haruhiko Siomi, Mikiko C. Siomi

**Affiliations:** 1 Department of Biological Sciences, Graduate School of Science, The University of Tokyo, Tokyo 113-0032, Japan;; 2 Department of Molecular Biology, Keio University School of Medicine, Tokyo 162-8582, Japan

**Keywords:** piRNA, PIWI, ping-pong cycle, *l(3)mbt*, CRISPR, *Drosophila*

## Abstract

In *Drosophila* germ cells, PIWI-interacting RNAs (piRNAs) are amplified through a PIWI slicer-dependent feed-forward loop termed the ping-pong cycle, yielding secondary piRNAs. However, the detailed mechanism remains poorly understood, largely because an ex vivo model system amenable to biochemical analyses has not been available. Here, we show that CRISPR-mediated loss of function of *lethal (3) malignant brain tumor* [*l(3)mbt*] leads to ectopic activation of the germ-specific ping-pong cycle in ovarian somatic cells. Perinuclear foci resembling nuage, the ping-pong center, appeared following *l(3)mbt* mutation. This activation of the ping-pong machinery in cultured cells will greatly facilitate elucidation of the mechanism underlying secondary piRNA biogenesis in *Drosophila*.

## Abstract (precis)

Sumiyoshi et al. show that CRISPR-mediated loss of function of *lethal (3) malignant brain tumor* [*l(3)mbt*] leads to ectopic activation of the germ-specific ping-pong cycle in ovarian somatic cells. Perinuclear foci resembling nuage, the ping-pong center, appeared following *l(3)mbt* mutation.

PIWI-interacting RNAs (piRNAs) form piRNA-induced silencing complexes (piRISCs) with PIWI proteins that repress transposons to maintain the integrity of the germline genome (Aravin et al. 2007; Brennecke et al. 2007; Ghildiyal and Zamore 2009; Juliano et al. 2011; Iwasaki et al. 2015). Somatic primary piRNA biogenesis in *Drosophila* has been extensively studied, mainly genetically and biochemically, using ovaries and a cultured ovarian somatic cell (OSC) line (Niki et al. 2006; Malone et al. 2009; Saito et al. 2009). According to the current model, piRNA precursors arising from piRNA clusters accumulate at specific granules, termed the Yb body and Dot COM, and are then processed at Yb bodies (Saito et al. 2010; Dennis et al. 2013; Murota et al. 2014). In parallel, Yb centralizes multiple primary piRNA factors to Yb bodies, including Armitage (Armi), Vreteno (Vret), Sister of Yb (SoYb), and Shutdown (Shu) (Iwasaki et al. 2015). Yb bodies are often surrounded by mitochondria (Murota et al. 2014). This unique spatial arrangement in the cytoplasm enables Zucchini (Zuc), an endonuclease located on the surface of mitochondria with its active site facing the cytosol, to process piRNA intermediates into mature piRNAs (Ipsaro et al. 2012; Nishimasu et al. 2012). Primary piRNAs form piRISCs with Piwi but not with the other PIWI members Aub and AGO3 because of their absence in somatic cells. Piwi–piRISCs translocated to the nucleus transcriptionally repress transposons (Iwasaki et al. 2015).

Germ cells in *Drosophila* express Aub, AGO3, and Piwi (Saito et al. 2006; Brennecke et al. 2007; Gunawardane et al. 2007; Nishida et al. 2007). Of those, Aub and Piwi, but not AGO3, are loaded with primary piRNAs derived from piRNA clusters (Li et al. 2009; Malone et al. 2009). Piwi–piRISCs are then transported to the nucleus, where they repress transposons transcriptionally. In contrast, Aub–piRISCs remain in the cytoplasm and repress transposons by cleaving their mRNAs in a slicer-dependent manner. This Aub slicer-dependent RNA cleavage gives rise to secondary piRNAs, which are loaded onto AGO3. Aub–piRISCs and AGO3–piRISCs then operate a slicer-dependent feed-forward loop termed the ping-pong cycle, yielding a substantial amount of secondary piRNAs (Brennecke et al. 2007; Gunawardane et al. 2007). However, the detailed mechanism of the germ-specific process remains poorly understood, largely because an ex vivo *Drosophila* model system amenable to biochemical analyses has not been available. BmN4 cells (Kawaoka et al. 2009) have been used to study the mechanism underlying the ping-pong cycle but are of lepidopteran origin. BmN4 cells express two PIWI members but lack a homolog of *Drosophila* Piwi.

In *Drosophila,* mutations in *lethal (3) malignant brain tumor* [*l(3)mbt*] generate malignant brain tumors with metastatic potential (Gateff et al. 1993; Janic et al. 2010) due to deregulation of *l(3)mbt* signature (MBTS) genes (Janic et al. 2010). Multiple MBTS genes encode proteins with germline functions, and mutations in these genes (for instance, *piwi*) rescue the brain tumor phenotype (Janic et al. 2010). MBTS genes with germline functions include ping-pong factors such as *aub* and *vasa* (Janic et al. 2010). A few small RNAs in *l(3)mbt* mutant brain tumors were annotated as piRNAs (Janic et al. 2010). These findings of *l(3)mbt* mutations resulting in ectopic acquisition of germline traits in the brain prompted us to examine whether depletion of *l(3)mbt* in OSCs initiates the ping-pong cycle and causes the accumulation of secondary piRNAs in cells.

In this study, we found that both RNAi- and CRISPR-mediated loss of function of *l(3)mbt* lead to ectopic expression of Aub, AGO3, and Vasa—the core factors necessary for operating the germ-specific ping-pong cycle—in OSCs. Deep sequencing and comparison of mRNAs in the CRISPR-mediated mutant OSCs (Δmbt-OSCs) and parental OSCs revealed that other ping-pong factors such as *qin* were up-regulated in Δmbt-OSCs. Both Aub and AGO3 copurified with piRNAs in Δmbt-OSCs. Deep sequencing these small RNA populations demonstrated that Aub- and AGO3-bound piRNAs show a typical ping-pong signature. Perinuclear foci resembling nuage, the ping-pong center (Brennecke et al. 2007; Gunawardane et al. 2007; Lim and Kai 2007; Malone et al. 2009), also appeared following *l(3)mbt* mutation. Depletion of Vasa in Δmbt-OSCs disrupted the ping-pong pathway and Aub localization to the nuage-like structure. Use of Δmbt-OSCs will greatly facilitate elucidation of the mechanism underlying secondary piRNA biogenesis and nuage formation in *Drosophila*.

## Results and Discussion

### Loss of *l(3)mbt* leads to ectopic expression of ping-pong factors in OSCs

OSCs express *l(3)mbt* (http://flybase.org/reports/FBrf0221009.html). We therefore treated OSCs with *l(3)mbt* siRNA duplexes (Supplemental Fig. S1A). The mRNA level of *l(3)mbt* was significantly decreased upon RNAi treatment (Supplemental Fig. S1B). In contrast, *ago3* and *vasa* were significantly up-regulated after *l(3)mbt* siRNA treatment, while changes in the level of *piwi* mRNA were negligible (Supplemental Fig. S1B). Western blotting detected AGO3, Vasa, and Aub in the RNAi-treated cells, while the levels of Piwi, Yb, Armi, and Krimper (Krimp) (Saito et al. 2009; Iwasaki et al. 2015; Sato et al. 2015) remained unchanged (Supplemental Fig. S1C).

**Figure 1.**
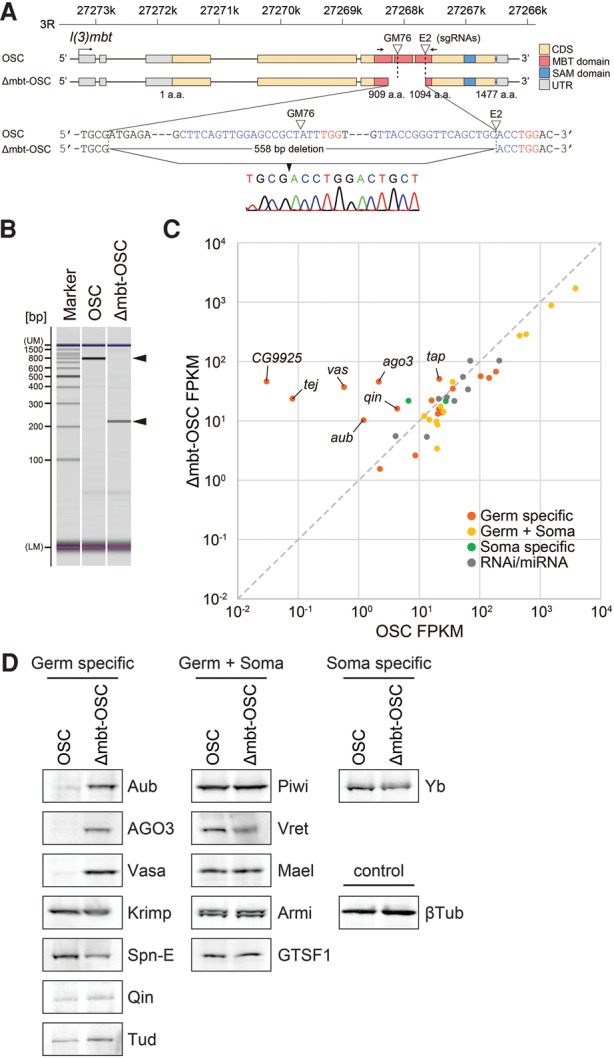
CRISPR-mediated generation of Δmbt-OSCs. (*A*) Genomic structure of *l(3)mbt* in OSCs and Δmbt-OSCs. sgRNAs GM76 and E2 targeting *l(3)mbt* exon 5 are indicated by arrowheads. Sequences at the *l(3)mbt* gene locus in OSCs and Δmbt-OSCs are also shown (sgRNA and PAM sequences are shown in blue and red, respectively). (CDS) Protein-coding DNA sequence; (MBT domain) malignant brain tumor domain; (SAM domain) sterile α motif domain; (UTR) untranslated region. (*B*) Genomic PCR fragments amplified with a primer set, shown by arrows in *A*, are indicated by arrowheads (OSC, 772 nt; Δmbt-OSC, 214 nt). (UM) Upper marker; (LM) lower marker. (*C*) Scatter plot comparing transcript abundance (mRNA-seq) of small RNA biogenesis factors in OSCs and Δmbt-OSCs. Classification of the factors to four clades—germ-specific, germ + soma, soma-specific, and RNAi/miRNA—was carried out with reference to Handler et al. (2013). (FPKM) Fragments per kilobase of exon per million mapped sequence reads. (*D*) Western blotting shows protein levels of piRNA factors in OSCs and Δmbt-OSCs. βTubulin (βTub) was detected as a loading control.

We next set out to ablate *l(3)mbt* function by editing the genome in OSCs using the CRISPR/Cas9 system. With reference to two mutant alleles, *l(3)mbt*^*GM76*^ and *l(3)mbt*^*E2*^ (Wismar et al. 1995; Yohn et al. 2003; Janic et al. 2010), small guide RNAs (sgRNAs) were designed to target exon 5, which contains MBT domains (Fig. 1A). The two mutant alleles have an effect on tumorigenesis in the fly brain (Wismar et al. 1995; Janic et al. 2010). A genomic PCR fragment amplified with a primer set flanking the sgRNA targeting sites was shortened after CRISPR/Cas9 treatment (Fig. 1B). Sequencing the fragment revealed that a 558-nucleotide (nt) deletion occurred at the gene locus (Fig. 1A). Full-length *l(3)mbt* mRNA was not detectable by quantitative RT–PCR (qRT–PCR) in the mutant cells (data not shown). We refer to these cells here as Δmbt-OSCs.

Deep sequencing and comparison of mRNAs in Δmbt-OSCs and the parental OSCs revealed that, in addition to *ago3* and *vasa*, other ping-pong factors such as *aub*, *qin*, *tapas*, and *tejas* (Specchia et al. 2010; Gangaraju et al. 2011; Czech et al. 2013; Handler et al. 2013; Patil et al. 2014; Iwasaki et al. 2015) were markedly up-regulated in Δmbt-OSCs (Fig. 1C; Supplemental Fig. S2A). Western blotting confirmed increased levels of Aub, AGO3, and Vasa in Δmbt-OSCs (Fig. 1D). The levels of Piwi and other piRNA factors examined, such as Yb and Armi (Iwasaki et al. 2015), were nearly identical between the two cell lines (Fig. 1D), although a few factors showed slight changes in their mRNA levels (Fig. 1C; Supplemental Fig. S2A). Germ-specific genes tended to be more sensitive to loss of *l(3)mbt* than germ + soma and soma-specific genes (Fig. 1C; Supplemental Fig. S2A). The appearance of Yb in Δmbt-OSCs suggests that CRISPR-mediated *l(3)mbt* loss had little effect on the elimination of somatic traits from the mutant cells. Focusing on MBTS genes revealed that many, if not all, were up-regulated in Δmbt-OSCs (Supplemental Fig. S2B). Among non-MBTS genes that are up-regulated by mutations in *mbt* genes other than MBTS (Janic et al. 2010), the highest up-regulation was observed for *ago3* (21.5-fold in Δmbt-OSCs). Results of transcriptome-wide analysis are summarized in Supplemental Tables S1 and S2. We confirmed that loss of *l(3)mbt* had very little effect on the proliferation rate of OSCs (Supplemental Fig. S2C).

### Loss of *l(3)mbt* activates the ping-pong cycle in OSCs

**Figure 2.**
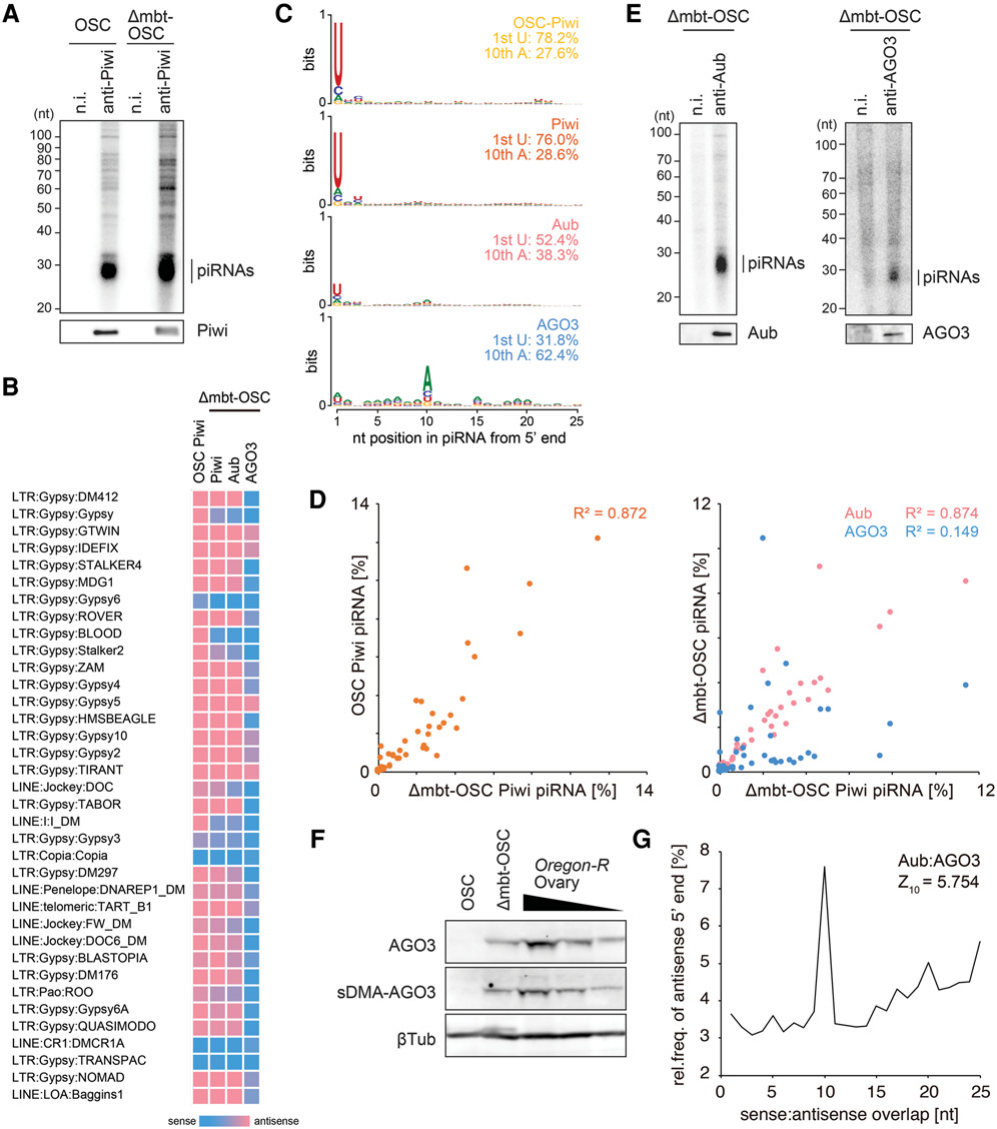
The ping-pong pathway in Δmbt-OSCs. (*A*) ^32^P-labeled piRNAs (*top* panel) associated with Piwi (*bottom* panel) in OSCs and Δmbt-OSCs. (n.i.) Nonimmune IgG. (*B*) Heat maps of piRNA strand bias. Strand bias of transposon-derived piRNAs in Piwi/Aub/AGO3 small RNA libraries from Δmbt-OSCs is shown. Strand bias of Piwi-bound piRNAs in parental OSCs (Ishizu et al. 2015) is also shown. (*C*) Sequence logos of Piwi/Aub/AGO3-bound piRNAs mapped to transposons in Δmbt-OSCs. Sequence logos of Piwi-bound piRNAs in parental OSCs (Ishizu et al. 2015) are also shown (OSC Piwi at the *top*). (*D*, *left*) Scatter plot showing the correlation of antisense Piwi-bound piRNA reads in OSCs (percentage; *Y*-axis) with those in Δmbt-OSCs (percentage; *X*-axis). (*Right*) The correlation of antisense Aub-bound (pink dots) and antisense AGO3-bound (blue dots) piRNA reads in Δmbt-OSCs (percentage; *Y*-axis) with antisense Piwi-bound piRNA reads in Δmbt-OSCs (percentage; *X*-axis) is also shown. Each dot corresponds to the ratio of piRNAs mapped to transposons (shown in *B*). (*E*) ^32^P-labeled piRNAs (*top* panel) associated with Aub and AGO3 (*bottom* panel) in Δmbt-OSCs. (n.i.) Nonimmune IgG. (*F*) Western blotting shows that AGO3 in Δmbt-OSCs is dimethylated, as in ovaries. (*G*) Depiction of the ping-pong signature between antisense Aub-bound piRNAs and sense AGO3-bound piRNAs, defined as the value at position 10 nt. Graphs indicate the relative frequency that a complementary piRNA exists with a 5′ end (*Y*-axis) at the indicated distance (*X-*axis).

To compare Piwi–piRNA loading between Δmbt-OSCs and OSCs, Piwi was immunoisolated from both lines, and extracted RNAs were ^32^P-labeled. piRNAs copurified with Piwi similarly from both cell lines (Fig. 2A). We then deep-sequenced Piwi-bound piRNAs in Δmbt-OSCs and compared the reads with those from OSCs (Fig. 2B–D; Supplemental Fig. S3A–D; Saito et al. 2009). This revealed that both sets of piRNAs show strong bias toward 1U and antisense orientation. The levels piRNAs from Δmbt-OSCs and parental OSCs mapped to each transposon were highly similar (*R*^2^ = 0.872). piRNAs were mapped similarly to *flamenco* (*flam*), a soma-specific single-strand piRNA cluster; *traffic jam* (*tj*), a genic piRNA source; and transposons such as *mdg1* (Brennecke et al. 2007; Malone et al. 2009; Robine et al. 2009; Saito et al. 2009). piRNAs corresponding to *42AB*, a germ-specific dual-strand piRNA cluster (Brennecke et al. 2007; Malone et al. 2009), were not contained in Piwi-bound piRNAs. This was expected because a component of the Rhino–Deadlock–Cutoff (RDC) complex, *cutoff*, which is necessary for producing piRNAs from *42AB* and other germ-specific dual-strand piRNA clusters (Mohn et al. 2014; Zhang et al. 2014), was undetected in both OSC lines (Supplemental Fig. S2A). Another component, *rhino*, whose expression was as low as *ago3* in OSCs, was even lower in Δmbt-OSCs, while *deadlock* was expressed moderately in both OSCs and Δmbt-OSCs (Supplemental Fig. S2A).

In Δmbt-OSCs, both Aub and AGO3 copurified with piRNAs (Fig. 2E). Deep sequencing these small RNA populations clarified that Aub-bound and AGO3-bound piRNAs show distinct features (Fig. 2B–D; Supplemental Fig. S3A–D). While Aub–piRNAs showed significant nucleotide bias toward 1U and were mostly antisense, AGO3–piRNAs had strong 10A and sense biases. The correlation between levels of Piwi-bound and AGO3-bound piRNAs mapped to each transposon was low (*R*^2^ = 0.149), while the correlation was significantly high between levels of Piwi-bound and Aub-bound piRNAs (*R*^2^ = 0.874). Both *flam*–piRNAs and *tj*–piRNAs were found with Aub but not with AGO3. *42AB*–piRNAs were not detected with Aub or AGO3. Additionally, plotting the distribution of Aub-bound and AGO3-bound piRNAs to transposons *DM412*, *DM297*, and *mdg1* indicated the production of piRNAs from the same coordinate but in the opposite orientation.

We previously showed that both Myc-Aub and Myc-AGO3 exogenously expressed in OSCs by transfection are loaded with primary piRNAs, including *flam*–piRNAs and *tj*–piRNAs, which are normally loaded onto endogenous Piwi (Olivieri et al. 2012; Sato et al. 2015). This emphasized the compatibility of AGO3 with primary piRNA loading. However, in Δmbt-OSCs, AGO3 avoided binding primary piRNAs; this was especially apparent when *tj*–piRNAs were compared (Supplemental Fig. S3C; Sato et al. 2015). In OSCs, Krimp sequesters unloaded AGO3 to Krimp bodies and blocks AGO3–primary piRNA loading (Olivieri et al. 2012; Sato et al. 2015). Krimp is crucial for AGO3 function in ovary germ cells (i.e., symmetrical dimethyl arginine [sDMA] modification and secondary piRNA association) (Sato et al. 2015; Webster et al. 2015); therefore, it would be expected that, in Δmbt-OSCs, AGO3 loading with secondary piRNAs might be under the control of Krimp. Indeed, AGO3 was sDMA-modified in Δmbt-OSCs, as in ovaries, while Myc-AGO3 in OSCs was sDMA-free (Fig. 2F; Supplemental Fig. S3E). However, this does not exclude the possibility that unknown proteins other than Krimp may also be involved in the regulation of AGO3 sDMA modification along with Krimp.

If AGO3-bound piRNAs were produced via the ping-pong cycle in an Aub slicer-dependent manner, a ping-pong signal—i.e., complementary overlap of 10 nt at the 5′ ends—should be observed between Aub-bound and AGO3-bound piRNAs. piRNA production by such a ping-pong cycle was evaluated, and a significant ping-pong signal was observed between Aub-bound and AGO3-bound piRNAs (Fig. 2G). These results, along with mapping data shown in Supplemental Figure S3D, further support the idea that AGO3-bound piRNAs are products of Aub slicer in the ping-pong pathway.

Piwi-bound primary piRNAs in OSCs involve a unique subset of piRNAs called phased piRNAs (Han et al. 2015; Mohn et al. 2015). Examination of piRNAs in Δmbt-OSCs by focusing on this trait revealed that phased piRNAs with *d* = 1 (this means that two piRNAs can be mapped right next to each other on the same genomic strand without a nucleotide gap between them) are particularly abundant in Piwi-bound piRNAs (*Z*_1_ = 22.3). This was comparable with Piwi-bound piRNAs (*Z*_1_ = 16.8) in OSCs (Supplemental Fig. S4A,B). *Z*_1_ scores of Aub-bound and AGO3-bound piRNAs in OSCs were 11.5 and 2.9, respectively (Supplemental Fig. S4A), suggesting that AGO3-bound piRNAs tend to exclude phased piRNAs (Han et al. 2015; Mohn et al. 2015). In contrast, piRNAs loaded onto Myc-AGO3 in OSCs (Sato et al. 2015) showed a significant phasing pattern (*Z*_1_ = 12.9) (Supplemental Fig. S4C). These results further support the notion that AGO3 in Δmbt-OSCs avoids binding primary piRNAs, in contrast to Myc-AGO3 in OSCs.

### Appearance of perinuclear foci resembling nuage in Δmbt-OSCs

**Figure 3.**
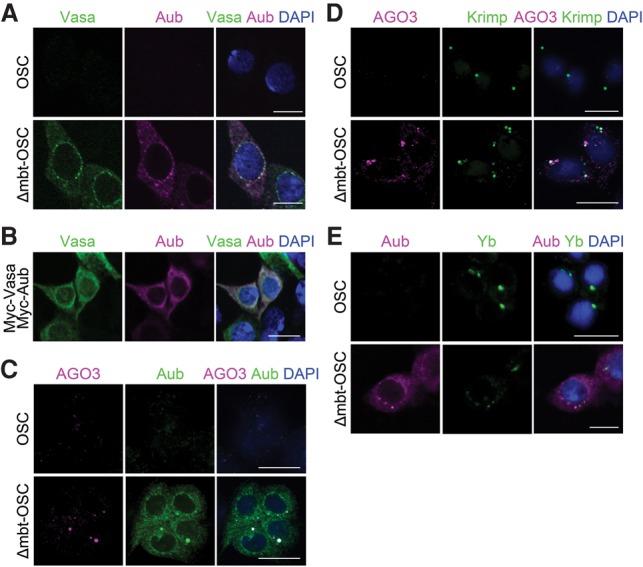
Nuage-like structures in Δmbt-OSCs. (*A*) Immunofluorescence shows that Aub and Vasa coincide at nuage-like structures in Δmbt-OSCs. (*B*) Coexpression of Myc-Aub with Myc-Vasa in OSCs by transfection. Immunofluorescence was performed using anti-Vasa and anti-Aub antibodies. (*C*) AGO3 colocalizes with Aub in Δmbt-OSCs. (*D*) AGO3 partially colocalizes with Krimp in Δmbt-OSCs. (*E*) Aub and Yb only occasionally coincide in Δmbt-OSCs. Bars, 10 µm.

Vasa, a germ-specific DEAD-box RNA helicase, plays a crucial role in the ping-pong cycle by displacing cleaved RNAs from Aub–piRISCs (or its counterpart, Siwi–piRISCs, in silkworms) in an ATP hydrolysis-dependent manner (Xiol et al. 2014; Nishida et al. 2015). Immunofluorescence in *Drosophila* ovaries showed that Aub, AGO3, and Vasa colocalize to nuage (Lim and Kai 2007; Malone et al. 2009; Iwasaki et al. 2015). Immunostaining of Δmbt-OSCs revealed that Aub and Vasa mostly coincide at perinuclear foci that look similar to nuage (Fig. 3A). Myc-Aub exogenously expressed in OSCs was uniformly distributed in the cytosol and did not form speckles (Sato et al. 2015). Signals of Myc-Vasa in OSCs were slightly stronger at the perinuclear region in the cytoplasm but not punctate (Fig. 3B). Myc-Aub coexpressed with Vasa in OSCs was distributed almost evenly in the cytoplasm (Fig. 3B). These results suggest that some factors whose expression is up-regulated by loss of *l(3)mbt* function promote Aub to form nuage-like structures with Vasa in Δmbt-OSCs.

Aub and AGO3 also colocalized to nuage-like structures (Fig. 3C). Krimp in OSCs sequesters unloaded AGO3 to Krimp bodies, which are generally present at a frequency of one per cell (Fig. 3D; Olivieri et al. 2012; Sato et al. 2015). However, in Δmbt-OSCs, AGO3-positive foci were more numerous than one per cell, and some could be superimposed with Aub-positive foci. This led us to examine the cellular localization of Krimp in Δmbt-OSCs, and we observed that co-localization of Krimp with AGO3 was apparent (Fig. 3D), as occurs in ovaries where Krimp normally accumulates at nuage. Krimp bodies are present only when piRNA biogenesis is defective because of mutations in piRNA factors such as *aub*. These results support the intriguing idea that Δmbt-OSCs likely recapitulate formation of the ping-pong center.

We next examined the spatial relationship between Yb and Aub-positive structures in Δmbt-OSCs. Aub and Yb signals only occasionally coincide (Fig. 3E), although the number of Yb bodies may be higher in Δmbt-OSCs compared with that in OSCs. These results suggest that primary and secondary piRNA biogenesis pathways may occur separately at Yb bodies and Aub-positive nuage-like foci, respectively, in Δmbt-OSCs.

**Figure 4.**
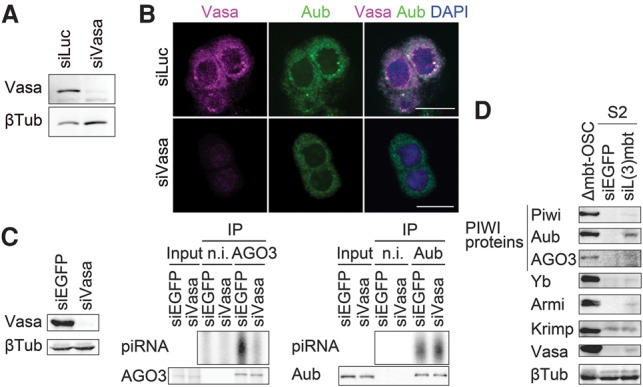
The effect of Vasa depletion in Δmbt-OSCs. (*A*) RNAi efficiently reduced the amount of Vasa in Δmbt-OSCs. siLuc was used as a negative control. (*B*) Depletion of Vasa in Δmbt-OSCs caused Aub to be scattered in the cytoplasm in Δmbt-OSCs. Bars, 10 µm. (*C*) AGO3, but not Aub, associates with fewer secondary piRNAs in Δmbt-OSCs upon Vasa RNAi treatment. siEGFP was used as a negative control. (n.i.) Nonimmune IgG. (*D*) Expression levels of piRNA factors in S2 cells upon *l(3)mbt* depletion by RNAi. dsEGFP (siEGFP) was used as a negative control.

In *vasa* mutant ovaries, Aub does not accumulate in the nuage and is evenly distributed in the cytosol of nurse cells (Lim and Kai 2007; Malone et al. 2009; Iwasaki et al. 2015). We therefore asked how depletion of Vasa affects Aub localization in Δmbt-OSCs. Treatment of Δmbt-OSCs with *vasa* siRNA duplexes caused Aub to be largely dispersed in the cytosol (Fig. 4A,B). Under such conditions, AGO3 was loaded with far fewer piRNAs, although Aub piRNA association remained largely unaffected (Fig. 4C). Thus, Vasa depletion disrupted the ping-pong pathway in Δmbt-OSCs, as expected.

### Loss of *l(3)mbt* fails to activate the ping-pong cycle in S2 cells

The *Drosophila* cell line S2 is of embryonic and somatic origin; therefore, PIWI proteins and piRNAs are below detection levels (Saito et al. 2006, 2009). *l(3)mbt* mRNA was detected in S2 cells by qRT–PCR (data not shown); therefore, we examined whether *l(3)mbt* knockdown has an impact on piRNA production in S2 cells. Upon RNAi, Aub was slightly up-regulated (Fig. 4D). However, very low levels of other factors, including Piwi, AGO3, and Vasa, were detected (Fig. 4D). Indeed, we failed to determine PIWI–piRNA loading by immunoprecipitation (data not shown). Although *Drosophila* brains and S2 cells are both nongonadal and somatic, the mechanism by which *l(3)mbt* controls downstream genes in these cells seems to be distinct.

In summary, our results show that CRISPR-mediated *l(3)mbt* knockout in OSCs leads to the ectopic expression of ping-pong factors, resulting in the accumulation of secondary piRNAs that are otherwise undetected in OSCs. In the mutant cells, AGO3-loaded secondary piRNAs were mostly 10A-biased and sense-oriented (Fig. 2; Supplemental Fig. S3). piRNA mapping revealed that the piRNAs originate from transposon mRNAs (Supplemental Fig. S3D). However, Piwi was expressed in Δmbt-OSCs to an extent similar to that in parental OSCs (Fig. 2A). Piwi-bound piRNAs were highly comparable in the two cell lines in all aspects, including piRNA origins and nucleotide and strand biases (Fig. 2; Supplemental Fig. S3). Thus, Piwi in Δmbt-OSCs should be similarly functional in repressing transposons compared with Piwi in OSCs. Indeed, Piwi was localized in the nucleus in both Δmbt-OSCs and OSCs (Supplemental Fig. S5A). The levels of transposon mRNAs were not drastically changed by loss of *l(3)mbt* (*R*^2^ = 0.988) (Supplemental Fig. S5B), suggesting that *l(3)mbt* is dispensable in transposon silencing in ovarian somas. The question then arises of where transposon mRNAs that give rise to sense AGO3-bound piRNAs came from in Δmbt-OSCs. Our previous study showed that overexpression of Myc-Aub in OSCs caused a slight but significant increase in the levels of *mdg1* and *DM297*, which are targets of Piwi in OSCs (Sato et al. 2015). This derepression of transposons could be because Myc-Aub competes with endogenous Piwi for primary piRNAs, impairing the Piwi-mediated transcriptional silencing activity. Indeed, AGO3-bound piRNAs included piRNAs originating from *mdg1* and *DM297* mRNAs (Fig. 2B). These results raise the intriguing idea that both transcriptional and post-transcriptional transposon silencing (i.e., the ping-pong cycle) occur in Δmbt-OSCs, mediated by nuclear Piwi–piRNA and cytoplasmic Aub–piRNA complexes, respectively, as in germ cells in fly ovaries. Thus, this newly made cell line, Δmbt-OSC, is the first cell line of any animal that accommodates both transcriptional and post-transcriptional silencing events and hence would also be useful in exploring the relationship of the two pathways.

## Materials and methods

### Establishment of Δmbt-OSCs

Gene knockout in OSCs using the CRIPSR/Cas9 system and isolation of knockout OSCs were performed essentially as described previously (Ishizu et al. 2015). In brief, OSCs were transfected with 0.2 μg of a plasmid expressing both pBS-Hsp70-Cas9 (Addgene, 46294) and the blasticidin resistance gene and 4 µg of two sgRNA-expressing plasmids (GM76 and E2) using Xfect transfection reagent (TaKaRa Clontech). After incubation for 24 h, blasticidin (Thermo Fisher Scientific, Inc., R210-01) was added to the culture medium at 50 µg/mL. The next day, 5.0 × 10^3^ cells were passaged in 3.5-cm dishes and allowed to grow in blasticidin-containing medium. During culturing, cells were washed with the medium to remove nonadherent dead cells. After 6–7 d of culture, 5.0 × 10^3^ cells were passaged in 6-cm dishes and allowed to grow in blasticidin-containing medium. After further incubation for a few days, colonies were picked and passaged in single wells of 24-well plates. The next day, these colonies were suspended with a Tip Strainer (Bel-Art) and allowed to grow to confluence. To detect the genomic deletion, genomic DNA was extracted using QuickExtract DNA extraction solution (Epicenter) following the manufacturer's protocol, and the genomic region flanking the CRISPR target site was amplified by PCR and then analyzed using MultiNa (Shimadzu).

## Supplementary Material

Supplemental Material
