# Supplementary material for: Loss of l(3)mbt leads to acquisition of the ping-pong cycle in Drosophila ovarian somatic cells
Source: Genes Dev. 2016 Jul 15;30(14):1617–22. doi: 10.1101/gad.283929.116 (PMC4973291; doi:10.1101/gad.283929.116)
Supplement: Supplemental Material [file supp_30_14_1617__index.html]

Supplemental Material 

# Loss of *l(3)mbt* leads to acquisition of the ping-pong cycle in *Drosophila* ovarian somatic cells

## Supplemental Material

**Files in this Data Supplement:**

- Supp\_Material.pdf
