# Supplementary material for: Loss of l(3)mbt leads to acquisition of the ping-pong cycle in Drosophila ovarian somatic cells
Source: Genes Dev. 2016 Jul 15;30(14):1617–22. doi: 10.1101/gad.283929.116 (PMC4973291; doi:10.1101/gad.283929.116)
Supplement: Supplemental Material [file supp_30.14.1617_Supp_Material.pdf]

Supplemental Material

Loss of *l(3)mbt* leads to acquisition of the ping-pong cycle in *Drosophila* ovarian somatic cells

Tetsutaro Sumiyoshi*, Kaoru Sato*, Hitomi Yamamoto, Yuka W. Iwasaki, Haruhiko Siomi and Mikiko C. Siomi
*These authors contributed equally to this work

Contents

Supplemental Materials and Methods
Figure legends for Supplemental Figures and Tables
Additional References
Supplemental Figures S1–S5
Supplemental Tables S1–S3

Supplemental Materials and Methods

Fly stock, cell culture and RNAi

Oregon-R was maintained at 25°C. Δmbt-OSCs and OSCs were grown at 26°C in culture medium prepared from Shields and Sang M3 Insect Medium (Sigma) supplemented with 0.6 mg/mL glutathione, 10% FBS, 10 mU/mL insulin, and 10% fly extract (Saito et al. 2009). Cell proliferation was monitored with the xCELLigence RTCA SP system (ACEA). For the RNAi of OSCs shown in Supplemental Fig. S1B, trypsinized cells (3×10^6 cells) were suspended in 100 μL of Solution V of the Cell Line Nucleofector Kit V (Amaxa Biosystems) together with 200 pmol of siRNA duplex. Transfection was conducted in electroporation cuvettes using a Nucleofector device 2b (Amaxa Biosystems). For the RNAi of Δmbt-OSCs and OSCs shown in Supplemental Fig. S1C, trypsinized cells (3×10^6 cells) were suspended in 20 μL of Solution SF of the Cell Line Nucleofector Kit SF (Amaxa Biosystems) together with 200–400 pmol of siRNA duplex. Transfection was conducted in a 96-well electroporation plate using a Nucleofector device 96-well Shuttle (Amaxa Biosystems). The transfected cells were transferred to fresh OSC medium and incubated at 26°C for 2–4 days for further experiments. S2 cells were grown at 26°C in Schneider's Insect medium (Sigma) supplemented with 10% fetal calf serum (Gibco), 50 μg/mL streptomycin, and 50 μg/mL penicillin (Sato et al. 2010). For RNAi in S2 cells, *l(3)mbt* cDNA was amplified by RT-PCR from OSC total RNA using gene-specific primers. It should be noted that the 5' end of each primer contained the T7 RNA polymerase promoter sequence. PCR products were purified using the FastGene Gel/PCR Extraction Kit (NIPPON Genetics) according to the manufacturer's instructions. Purified PCR products were used to produce dsRNAs using a T7-Scribe Standard RNA IVT Kit (CELLSCRIPT). The resultant RNAs were purified according to the manufacturer's instructions, heated for 5 min at 95°C, and then left to cool to room temperature. Then, 5×10^6 cells were suspended with 5 μg of dsRNA and incubated for 4 days at 26°C. The siRNAs and PCR primers used in this study are summarized in Supplemental Table S3.

Plasmid constructs and OSC transfection

Construction of the Myc-Aub, Myc-AGO3, and Myc-EGFP vectors used in this

study was described previously (Sato et al. 2015). To construct a Myc-Vasa expressing plasmid, a full-length *vasa* cDNA was amplified by RT-PCR and subcloned into pAcM under the control of the actin 5C promoter. SgRNA-expressing vectors were produced by inverse PCR using pU6-BbsI-chiRNA (Addgene, 45946). Primers are summarized in Supplemental Table S3. OSC transfection was performed using Xfect Transfection Reagent (Clontech) as reported previously (Saito et al. 2009; Saito et al. 2010; Sato et al. 2015) or ScreenFect A (Wako) following the manufacturer' s protocol (in Fig. 4B).

Immunoprecipitation

Immunoprecipitation was performed as previously described (Sato et al. 2015). In brief, immunoprecipitation of Piwi and Aub complexes from Δmbt-OSCs was performed using anti-Piwi (Saito et al. 2006) and anti-Aub (Nishida et al. 2007) antibodies in NP40 buffer. Immunoprecipitation of AGO3 complexes was performed using an anti-AGO3 antibody (a kind gift from Dr. Dahua Chen, Chinese Academy of Sciences, China) in RIPA binding buffer.

Western blotting

Western blotting was performed in principle as described previously (Miyoshi et al. 2005). Anti-Piwi, anti-Aub, anti-AGO3, anti-Krimp, anti-Spn-E, anti-Tud, anti-Vret, anti-Mael, anti-Armi, anti-GTSF1, anti-Yb, and anti-sDMA-AGO3 antibodies have been described previously (Saito et al. 2006; Nishida et al. 2007; Gunawardane et al. 2007; Nishida et al. 2009; Saito et al. 2010; Sato et al. 2011; Nagao et al. 2011; Ohtani et al. 2014; Sato et al. 2015). The anti-Qin antibody was kindly gifted by Dr. T. Kai. The mouse monoclonal anti-Vasa antibody was raised specifically against the full-length protein, essentially as described previously (Ishizuka et al. 2002) and was purified from the culture supernatant of hybridoma cells under standard procedures using Thiophilic-Superflow Resin (BD Biosciences). The anti-βTub antibody was obtained from the Developmental Studies Hybridoma Bank and used at a dilution of 1:1,000. Anti-mouse IgG, HRP-linked antibody (MP Biomedicals, 55558), and anti-rabbit IgG, HRP-linked antibody (CST) were used at dilutions of 1:5,000 and 1:1,000, respectively.

Immunofluorescence

Immunostaining of Δmbt-OSCs and OSCs was performed as previously described (Sato et al. 2015). The anti-Myc (Sigma C3956) antibody was used at 1:500. Anti-Piwi, anti-Aub, and anti-Krimp, anti-AGO3, and anti-Yb antibodies have been described previously (Saito et al. 2010; Sato et al. 2015; Nishida et al. 2007; Gunawardane et al. 2007). The anti-Vasa antibody was used at 1:250. Alexa Fluor488-conjugated anti-mouse IgG, Alexa Fluor546 conjugated anti-mouse IgG1, and Alexa Fluor488-conjugated anti-mouse IgG2a (Molecular Probes) were used as secondary antibodies. Cells were mounted in VECTASHIELD with DAPI (4',6-diamidino-2-phenylindole dihydrochloride) (Vector). All images were collected using a Zeiss LSM510 laser scanning microscope (Carl Zeiss). Image processing and annotation were performed using Adobe Photoshop (Adobe), ZEN (Carl Zeiss), and ImageJ software (National Institute of Health).

qRT-PCR

qRT-PCR was performed as previously described (Sato et al. 2015). In brief, total RNAs were isolated using ISOGEN (Nippon Gene) according to the manufacturer's instructions. Total RNAs were treated with DNase to eliminate DNA contamination. Total RNA (1 μg) was annealed with an oligo-dT primer and reverse transcribed using a Transcriptor First strand cDNA Synthesis Kit (Roche) according to the manufacturer's instructions. The resulting cDNAs were amplified with StepOnePlus (Applied Biosystems) using SYBR Premix Ex Taq (TaKaRa). The primer sets used are shown in Supplemental Table S1. The amplification efficiency of a qPCR reaction was calculated based on the slope of the standard curve. After confirming the amplification efficiency values (between 95% and 105%), relative steady-state RNA levels were determined from the threshold cycle for amplification.

Visualization of small RNAs

Visualization of small RNAs was performed as previously described (Sato et al. 2015). In brief, for RNA labeling with radioisotopes, total RNAs and

immunopurified RNAs were dephosphorylated with CIP (NEB), and then labeled with γ-^{32}P-ATP using T4 polynucleotide kinase (TaKaRa). The radiolabeled RNAs were separated on a denaturing polyacrylamide gel. The signal was detected using a Typhoon FLA 9500 (GE Healthcare).

Preparation of total RNA libraries and bioinformatic analysis

rRNAs were removed from isolated total RNAs using a Ribo-Zero rRNA Removal Kit (Human/Mouse/Rat) (Illumina). Total mRNA libraries were prepared using a TruSeq Stranded mRNA HT Sample Prep Kit (Illumina) according to the manufacturer's instructions and sequenced using a HiSeq 2500 (Illumina). Paired-end sequence reads were split into forward and reverse reads and mapped separately on the *Drosophila* genome (Dm3, BDGP Release 5), and then annotated to *Drosophila* genes defined in the UCSC Genome Browser (FlyBase) using Cuffcompare. Differential expression analysis of mRNA-seq expression profile was performed using edgeR package in R. The lists of differentially expressed genes ($p < 0.05$) were applied for functional annotation (gene ontology) analysis using DAVID. Paired-end sequence reads were also mapped to the transposon consensus sequences (Senti et al. 2015). To compare mRNA abundance between two libraries, FPKM (fragments per kilobases of exons per million mapped reads) normalization was performed.

Preparation of small RNA libraries and bioinformatic analysis

Cloning and analysis of small RNA libraries were performed essentially as previously described (Sato et al. 2015). In brief, 20–30 nt small RNA libraries were prepared manually based on the manufacturer's instructions of a TruSeq Small RNA Sample Prep Kit (Illumina), and then sequenced using MiSeq (Illumina). Reads perfectly mapped on the *Drosophila* genome (Dm3, BDGP Release 5) were used. Alignments overlapping with rRNAs, tRNAs and snoRNAs were removed, and then small RNAs in the range of 24–30 nt were selected. The mapped reads for immunoprecipitation libraries do not reflect actual cellular abundance and, therefore, we performed a relative comparison of how piRNA sequences are loaded on to each PIWI protein. For transposon analysis, we mapped all 24–30 nt small RNAs to *Drosophila* transposable

element sequences obtained from the UCSC Genome Browser (RepeatMasker). Sense/antisense strand bias, sequence logo, and ping-pong signature on each transposon or all transposons were analyzed as previously described (Sato et al. 2015). To calculate ping-pong Z_{10} scores, overlaps at position 1–9 and 11–25 nt were used as background. Phasing analysis was performed as previously described (Han et al. 2015). The score for a 3'-to-5' distance was calculated by Σminimal (M*i*, N*i*+*x*); M*i* is the number of upstream reads whose 3' ends are located at position *i*, and N*i*+*x* is the number of downstream reads whose 5' ends are located at position *i*+*x*. To calculate the ping-pong Z_1 score, overlaps at position 0 and 2–50 nt were used as background.

Deep sequencing datasets have been deposited in the DDBJ Sequence Read Archive under the accession number DRA004265.

Figure legends for Supplemental Figures and Tables

Supplemental Figure S1. Ectopic expression of piRNA factors in *l(3)mbt*-depleted OSCs. (**A**) Genomic structure of the *l(3)mbt* gene and siRNA target sites. (**B**) qRT-PCR shows that *vasa* and *ago3* were up-regulated in OSCs upon *l(3)mbt* RNAi treatment. In contrast, the *piwi* mRNA level was unchanged by *l(3)mbt* depletion. *rp49* was used as an internal control. N = 3; error bars indicate SEM. (**C**) Western blotting confirms up-regulation of Aub, AGO3, and Vasa by *l(3)mbt* depletion in OSCs. βTubulin (βTub) was employed as a loading control.

Supplemental Figure S2. Expression of piRNA factors and MBTS/non-MBTS genes in Δmbt-OSCs. (**A**) Transcript abundance of small RNA biogenesis factors (germ specific, germ + soma, and soma specific) (Handler et al. 2013) in OSCs and Δmbt-OSCs measured by mRNA-seq. FPKM: Fragments per kilobase of exon per million mapped sequence reads. (**B**) Left: Scatter plot comparing transcript abundance (mRNA-seq) of MBTS and non-MBTS genes in OSCs and Δmbt-OSCs. Classification of MBTS and non-MBTS genes was carried out with reference to Janic *et al.* (Janic et al. 2010). Right: Transcript abundance of MBTS and non-MBTS genes in OSCs and Δmbt-OSCs measured by mRNA-seq. (**C**) Real-time monitoring of cell proliferation. Cell-sensor impedance was expressed as an arbitrary unit called the Cell index. The Cell index values were plotted against time.

Supplemental Figure S3. Characterization of piRNAs in Δmbt-OSCs. (**A**) Pie charts summarizing the annotation of small RNA populations in total small RNAs, and Piwi/Aub/Ago3-bound piRNAs. (**B**) Small RNA size profiles for total small RNAs, and Piwi/Aub/Ago3-bound piRNAs. Sequences corresponding to rRNA, tRNA, snRNA, and snoRNA were omitted before analysis. (**C**) Mapping of Piwi/Aub/Ago3-bound piRNAs in Δmbt-OSCs and Piwi-bound piRNAs in parental OSCs (Ishizu et al. 2015) to *flam*, *tj*, and *42AB*. (**D**) Mapping of Piwi/Aub/Ago3-bound piRNAs in Δmbt-OSCs to *DM412*, *DM297,* and *mdg1* transposons. Mapping of Piwi-bound piRNAs in OSCs was also shown as a

reference (Ishizu et al. 2015). (**E**) Myc-AGO3 overexpressed in OSCs by transfection is sDMA free. Asterisks indicate background signals.

Supplemental Figure S4. Characterization of phased piRNAs in Δmbt-OSCs. (**A**) Analyses of phased piRNAs in total small RNAs, and Piwi/Aub/AGO3-bound piRNAs in Δmbt-OSCs. The distance between the 3' end of the upstream piRNA and the 5' end of downstream piRNAs on the same genomic strand was analyzed. (**B**) Comparison of phased piRNAs in Piwi-bound piRNAs in OSCs (Ishizu et al. 2015) and Δmbt-OSCs. (**C**) Analyses of phased piRNAs in AGO3-bound piRNAs in Δmbt-OSCs and Myc-AGO3-bound piRNAs in OSCs (Sato et al. 2015).

Supplemental Figure S5. Piwi maintains its transposon silencing functions in Δmbt-OSCs. (**A**) Immunofluorescence reveals that Piwi is localized in the nucleus of Δmbt-OSCs. Scale bars indicate 10 μM. (**B**) Scatter plot comparing transcript abundance (mRNA-seq) of transposons in OSCs and Δmbt-OSCs.

Supplemental Table S1. List of Gene Ontology terms of the biological process category associated with differentially expressed genes in OSCs and Δmbt-OSCs.

Supplemental Table S2. List of differentially expressed genes in OSCs and Δmbt-OSCs.

Supplemental Table S3. List of siRNAs and primers. RNA is shown in italics.

Additional References

Ishizuka A, Siomi MC, Siomi H. 2002. A *Drosophila* fragile X protein interacts with components of RNAi and ribosomal proteins. *Genes Dev* **16:** 2497–2508.

Miyoshi K, Tsukumo H, Nagami T, Siomi H, Siomi MC. 2005. Slicer function of *Drosophila* Argonautes and its involvement in RISC formation. *Genes Dev* **19:** 2837–2848.

Nagao A, Sato K, Nishida KM, Siomi H, Siomi MC. 2011. Gender-specific hierarchy in nuage localization of PIWI-interacting RNA factors in *Drosophila*. *Front Genet* **2:** 55.

Nishida KM, Okada TN, Kawamura T, Mituyama T, Kawamura Y, Inagaki S, Huang H, Chen D, Kodama T, Siomi H, et al. 2009. Functional involvement of Tudor and dPRMT5 in the piRNA processing pathway in *Drosophila* germlines. *EMBO J* **28:** 3820–3831.

Ohtani H, Iwasaki YW, Shibuya A, Siomi H, Siomi MC, Saito K. 2013. DmGTSF1 is necessary for Piwi-piRISC-mediated transcriptional transposon silencing in the *Drosophila* ovary. *Genes Dev* **27:** 1656–1661.

Sato K, Nishida KM, Shibuya A, Siomi MC, Siomi H. 2011. Maelstrom coordinates microtubule organization during *Drosophila* oogenesis through interaction with components of the MTOC. *Genes Dev* **25:** 2361–2373.

Senti KA, Jurczak D, Sachidanandam R, Brennecke J. 2015. piRNA-guided slicing of transposon transcripts enforces their transcriptional silencing via specifying the nuclear piRNA repertoire. *Genes Dev* **29:** 1747–1762.

Supplemental Fig. S1

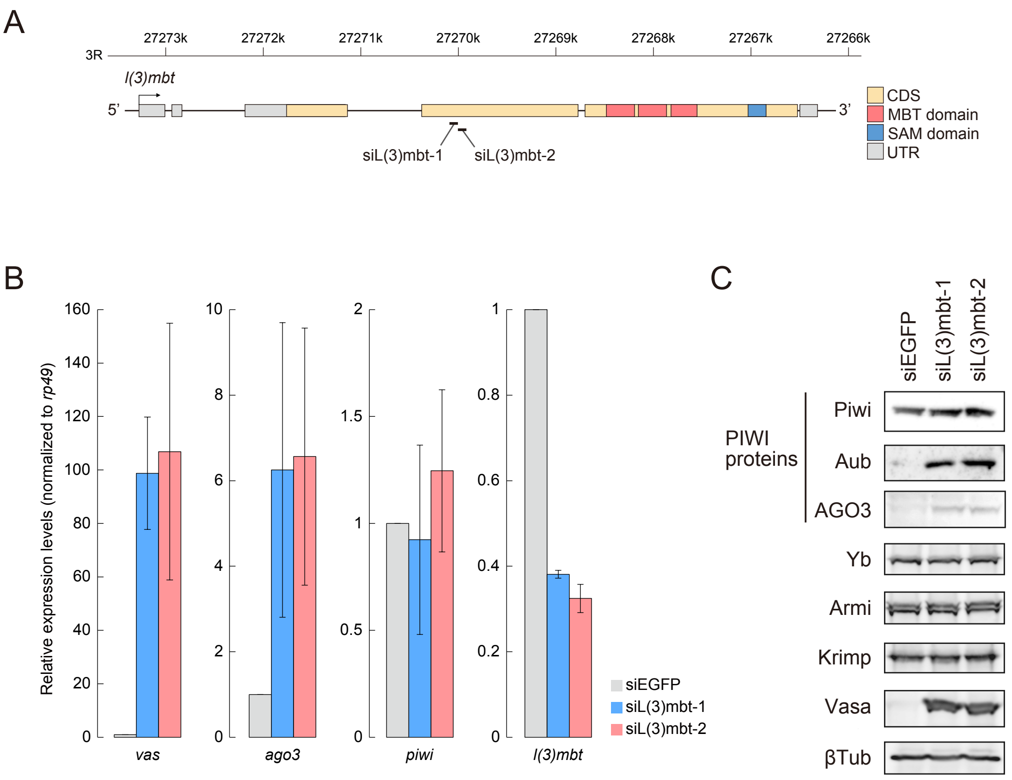

Supplemental Fig. S2

A

	OSC [FPKM]	Δmbt-OSC [FPKM]
**Germ specific**		
UAP56	184.58	67.59
spn-E	142.42	53.02
krimp	102.93	56.68
tud	35.75	34.89
csul	23.63	13.86
tap	21.34	51.13
papi	21.14	15.88
vls	20.24	13.22
del	15.94	22.25
BoYb	8.58	2.62
qin	4.31	16.15
rhi	2.23	1.56
ago3	2.14	45.96
aub	1.20	10.30
vas	0.57	37.22
tej	0.08	23.84
CG9925	0.03	46.27
squ	0	0
cuff	0	0

	OSC [FPKM]	Δmbt-OSC [FPKM]
**Germ + Soma**		
shu	3798.68	1721.88
hsp83	1516.88	884.65
armi	582.34	289.24
piwi	454.98	273.26
mael	35.66	45.46
minotaur	25	14.29
vreteno	22.56	17.52
zuc	19.88	8.69
gasz	19.6	3.42
CG9754	19.17	9.77
gtsf1	14.63	10.5
egg	12.06	12.05
Pimet	0	12.46
**Soma specific**		
SoYb	27.33	21.64
fs(1)Yb	6.64	21.79

B

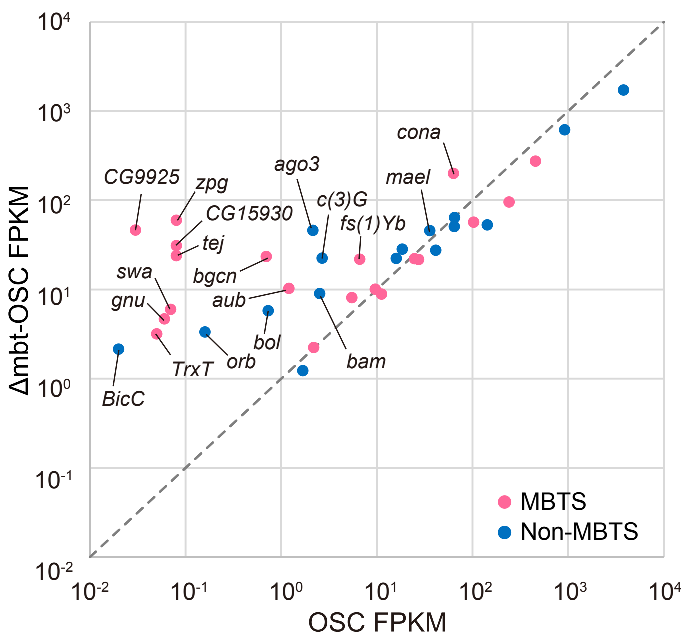

	OSC	Δmbt-OSC
**MBTS**		
piwi	454.98	273.26
RpS5b	241.47	95.74
krimp	102.93	56.68
cona	63.21	199.68
CG31755	27.33	21.64
CG7795	24.80	22.06
hdm	11.23	8.88
fus	9.64	10.06
fs(1)Yb	6.64	21.79
mia	5.48	8.07
topi	2.18	2.24
aub	1.20	10.30
bgcn	0.70	23.33
CG15930	0.08	31.00
tej	0.08	23.84
zpg	0.08	59.59
swa	0.07	5.96
gnu	0.06	4.66
TrxT	0.05	3.18
CG9925	0.03	46.27
dhd	0	65.1
CG32313	0	4.54
γTUB37C	0	1.97
nos	0	17.66
stil	0	0.19
Ptx	0	0

	OSC	Δmbt-OSC
**Non-MBTS**		
shu	3798.68	1721.88
CG7194	918.29	614.28
spn-E	142.42	53.02
RpS19b	64.99	63.98
mre11	64.59	50.86
loki	41.26	27.42
mael	35.66	45.46
ovo	18.47	28.19
del	15.94	22.25
c(3)G	2.68	22.44
bam	2.52	9.00
ago3	2.14	45.96
png	1.68	1.23
bol	0.73	5.79
orb	0.16	3.34
BicC	0.02	2.14
vis	0	1.40
tor	0	4.24
Mst57Db	0	0
Mst77F	0	0
squ	0	0
CG40115	0	0

C

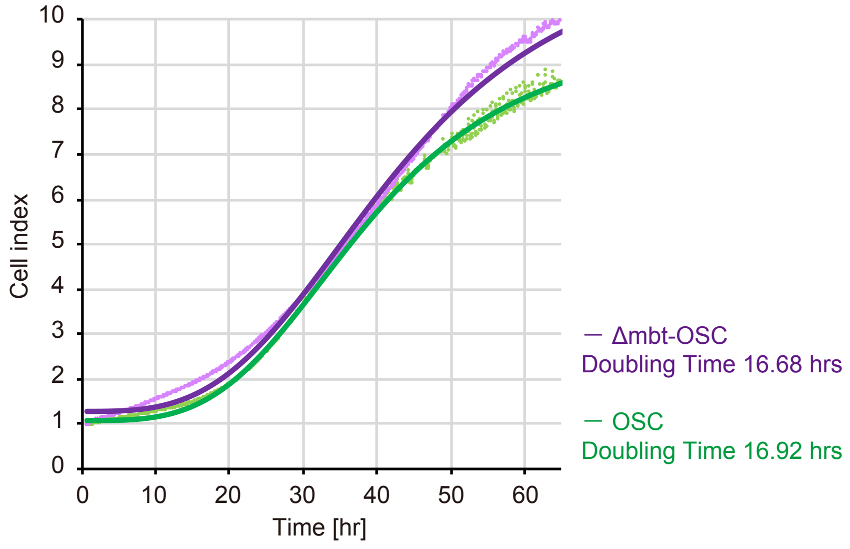

Supplemental Fig. S3

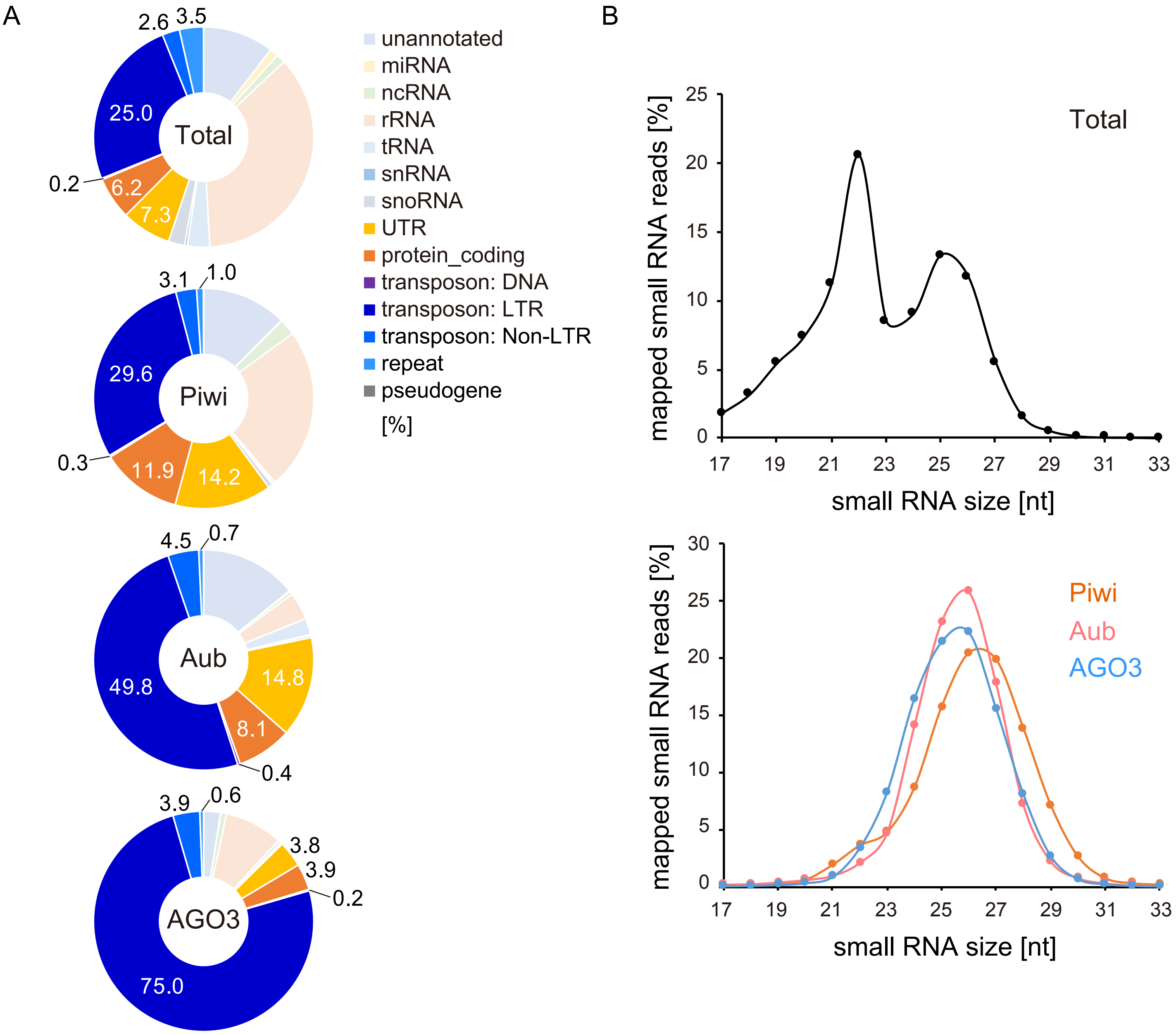

Supplemental Fig. S3 (continued)

C

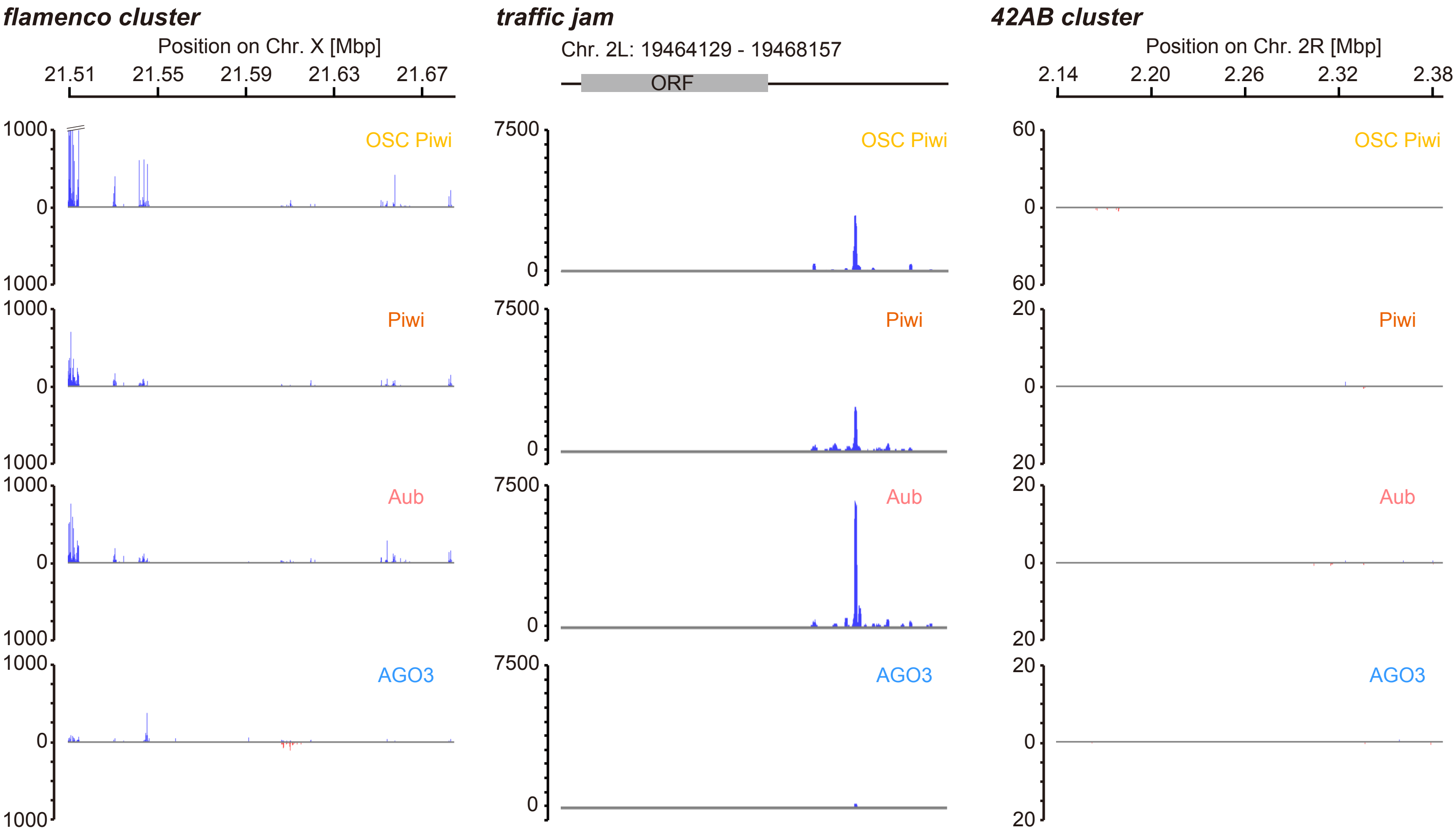

D

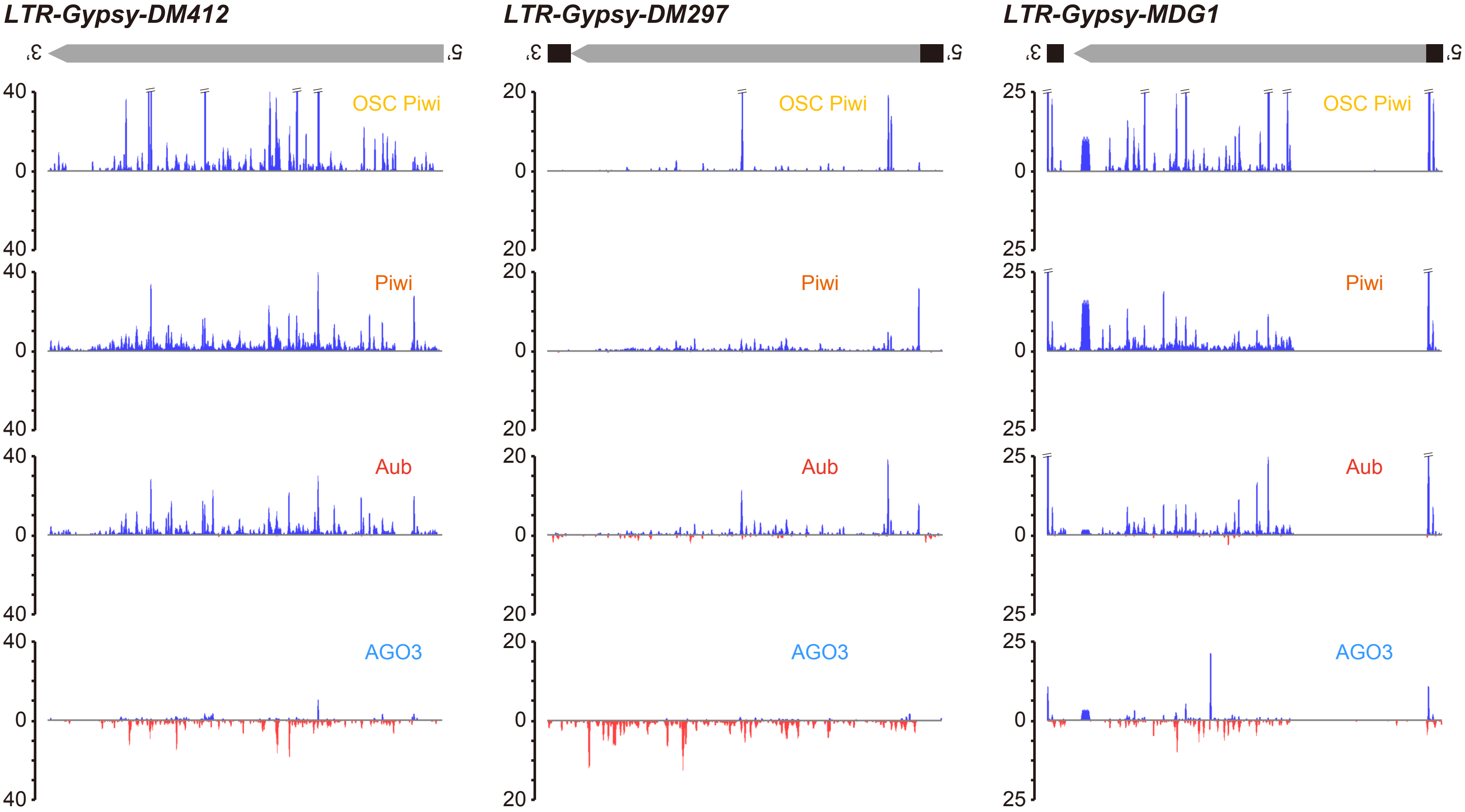

Supplemental Fig. S3 (continued)

E

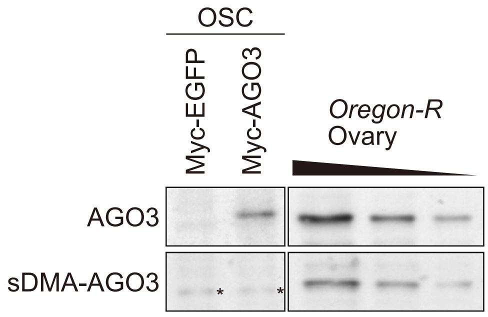

Supplemental Fig. S4

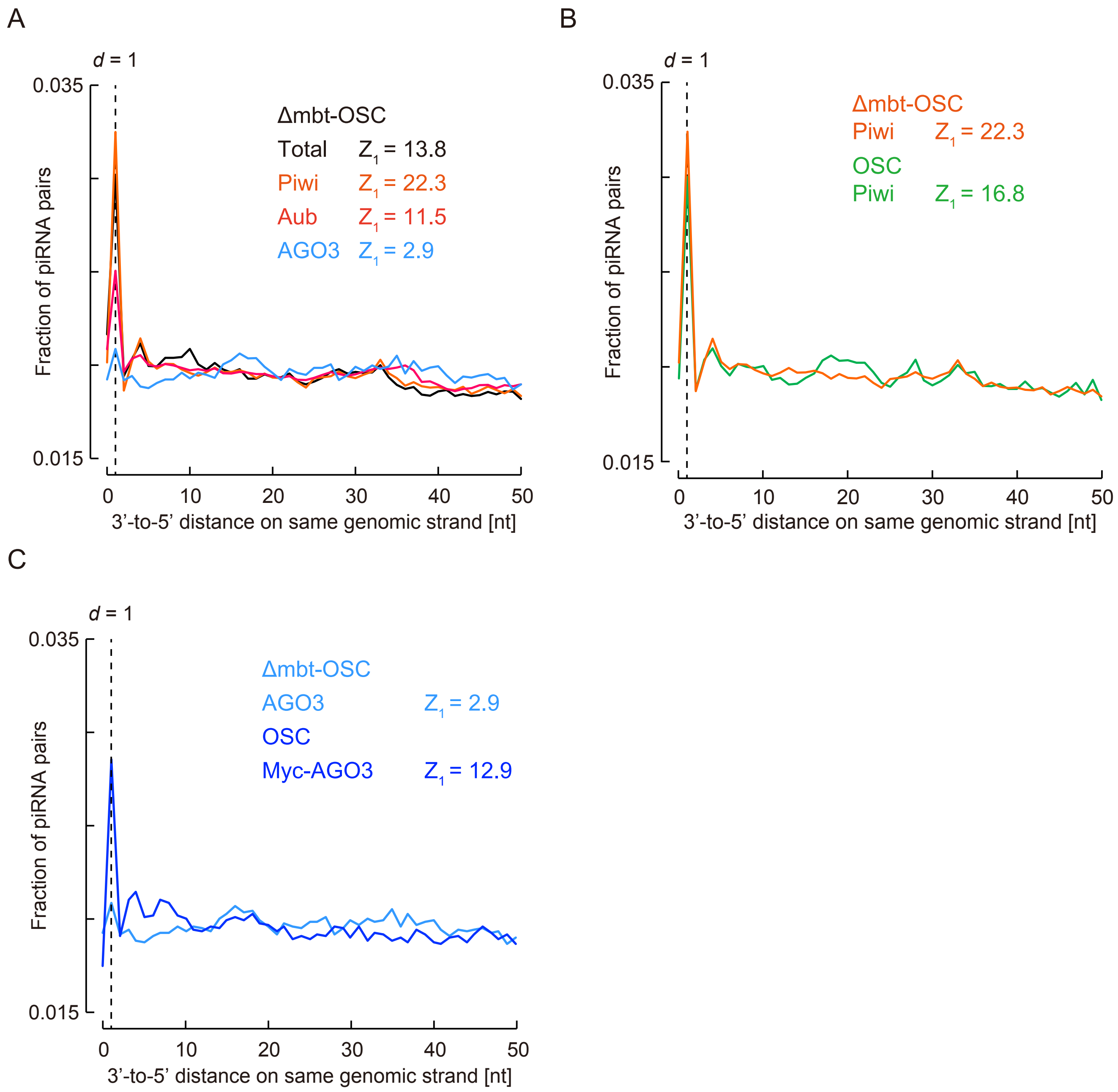

Supplemental Fig. S5

A

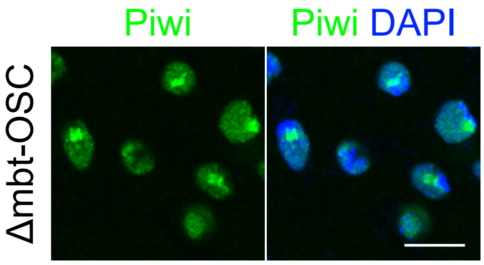

B

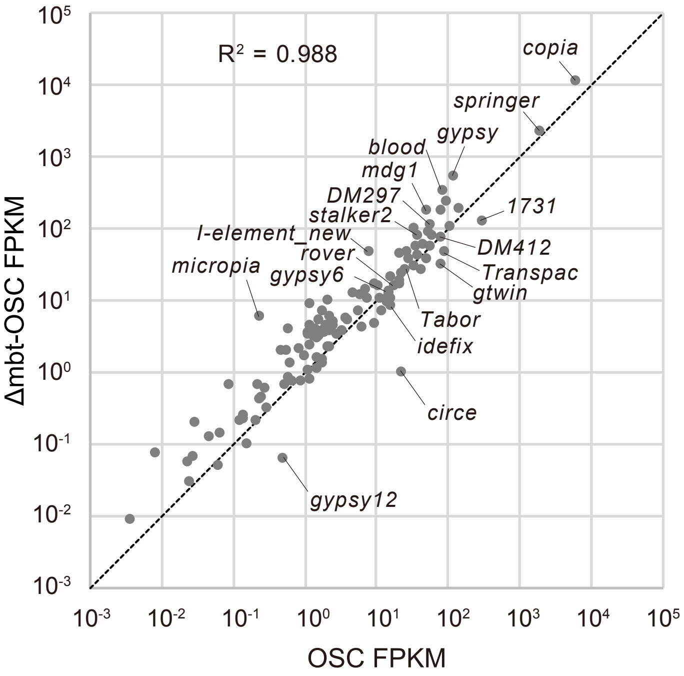

Supplemental Table S1. List of Gene Ontology terms of the biological process category associated with differentially expressed genes in OSCs and Δmbt-OSCs.

OSC < Δmbt-OSC

Term	PValue
GO:0055114~oxidation reduction	1.41E-04
GO:0007155~cell adhesion	0.004
GO:0045727~positive regulation of translation	0.006
GO:0006026~aminoglycan catabolic process	0.008
GO:0022610~biological adhesion	0.009
GO:0046012~positive regulation of oskar mRNA translation	0.009
GO:0000272~polysaccharide catabolic process	0.010
GO:0006928~cell motion	0.011
GO:0042461~photoreceptor cell development	0.013
GO:0060429~epithelium development	0.014
GO:0009792~embryonic development ending in birth or egg hatching	0.019
GO:0007398~ectoderm development	0.019
GO:0042462~eye photoreceptor cell development	0.021
GO:0048729~tissue morphogenesis	0.022
GO:0010648~negative regulation of cell communication	0.023
GO:0007422~peripheral nervous system development	0.025
GO:0001700~embryonic development via the syncytial blastoderm	0.026
GO:0007354~zygotic determination of anterior/posterior axis, embryo	0.028
GO:0034329~cell junction assembly	0.031
GO:0046530~photoreceptor cell differentiation	0.034
GO:0007127~meiosis I	0.034
GO:0006032~chitin catabolic process	0.034
GO:0032270~positive regulation of cellular protein metabolic process	0.034
GO:0051247~positive regulation of protein metabolic process	0.034
GO:0042067~establishment of ommatidial polarity	0.037
GO:0007164~establishment of tissue polarity	0.038
GO:0002009~morphogenesis of an epithelium	0.038
GO:0007561~imaginal disc eversion	0.039
GO:0048598~embryonic morphogenesis	0.039
GO:0009952~anterior/posterior pattern formation	0.042
GO:0008544~epidermis development	0.043
GO:0001754~eye photoreceptor cell differentiation	0.044
GO:0030178~negative regulation of Wnt receptor signaling pathway	0.045
GO:0001745~compound eye morphogenesis	0.045
GO:0009880~embryonic pattern specification	0.046
GO:0048610~reproductive cellular process	0.047
GO:0007411~axon guidance	0.048
GO:0016477~cell migration	0.050
GO:0048134~germ-line cyst formation	0.050

OSC > Δmbt-OSC

Term	PValue
GO:0000022~mitotic spindle elongation	0.010
GO:0051231~spindle elongation	0.011
GO:0006412~translation	0.015
GO:0007052~mitotic spindle organization	0.017
GO:0007051~spindle organization	0.038
GO:0000278~mitotic cell cycle	0.041
GO:0006350~transcription	0.048
GO:0009303~rRNA transcription	0.050
GO:0008272~sulfate transport	0.050

Supplemental Table S2. List of differentially expressed genes in OSCs and Δmbt-OSCs.

OSC > Δmbt-OSC

ID	Symbol	OSC-FPKM	Δmbt-OSC FPKM	OSC/Δmbt-OSC FC	PValue
CG12641	CG12641	1698.57	0	13.51	1.91E-18
CG7075	Ntl	528.05	0	11.83	3.00E-14
CG5277	Ip259	485.04	0	11.70	5.99E-14
CG9336	CG9336	283.61	0	10.93	4.54E-12
CG41623	CG41623	149.29	0	10.00	6.74E-10
CG6128	Fuca	115.98	0	9.64	4.46E-09
CG13240	l(2)35Di	74.05	0	8.99	1.11E-07
CG6842	CG6842	68.92	0	8.89	1.81E-07
CG30481	mRpL53	62.77	0	8.76	3.40E-07
CG32701	l(1)G0320	58.97	0	8.67	5.15E-07
CG17084	mthl9	56.07	0	8.59	7.18E-07
CG30022	CG30022	47.81	0	8.36	2.00E-06
CG3192	CG3192	47.57	0	8.36	2.06E-06
CG7739	CG7739	42.84	0	8.21	3.96E-06
CG3999	CG3999	41.78	0	8.17	4.61E-06
CG5442	SC35	40.63	0	8.13	5.46E-06
CG17397	MED21	39.73	0	8.10	6.25E-06
CG3988	gammaSnap	39.45	0	8.09	6.52E-06
CG12279	CG12279	38.87	0	8.07	7.13E-06
CG5385	CG5385	37.75	0	8.03	8.48E-06
CG6289	CG6289	35.63	0	7.94	1.19E-05
CG6249	Csl4	35.6	0	7.94	1.20E-05
CG8613	CG8613	34.31	0	7.89	1.48E-05
CG6677	ash2	34.12	0	7.88	1.53E-05
CG8264	Bx42	33.23	0	7.84	1.78E-05
CG31478	mRpL9	32.37	0	7.80	2.07E-05
CG11753	CG11753	31.55	0	7.77	2.39E-05
CG30159	CG30159	31.19	0	7.75	2.55E-05
CG9362	CG9362	30.7	0	7.73	2.79E-05
CG5189	CG5189	30.6	0	7.72	2.84E-05
CG9240	CG9240	30.18	0	7.70	3.06E-05
CG5403	retn	28.4	0	7.62	4.28E-05
CG9006	Egm	26.66	0	7.53	6.01E-05
CG5013	CG5013	26.5	0	7.52	6.20E-05
CG30051	CG30051	26.26	0	7.50	6.51E-05
CG4199	CG4199	26.19	0	7.50	6.60E-05
CG5575	ken	25.68	0	7.47	7.32E-05
CG9378	Rlc1	25.13	0	7.44	8.20E-05
CG11071	CG11071	24.43	0	7.40	9.49E-05
CG4880	CG4880	24.22	0	7.39	9.92E-05
CG13162	ana3	24.03	0	7.38	1.03E-04
CG9889	yellow-d	155.07	0.57	7.37	4.09E-09
CG2098	ferrochelatase	22.79	0	7.30	1.35E-04
CG30105	CG30105	22.34	0	7.27	1.49E-04
CG7694	CG7694	21.29	0	7.20	1.90E-04
CG42359	CG42359	20.31	0	7.14	2.38E-04
CG1584	Orc6	19.91	0	7.11	2.62E-04
CG42455	CG42455	19.64	0	7.09	2.80E-04
CG8333	HLHmgamma	19.58	0	7.08	2.84E-04
CG14581	CG14581	19.32	0	7.06	3.02E-04
CG5649	kin17	19.16	0	7.05	3.14E-04
CG8427	SmD3	18.77	0	7.02	3.46E-04
CG33228	CG33228	18.69	0	7.02	3.53E-04
CG6903	CG6903	18.23	0	6.98	3.96E-04
CG8498	CG8498	17.65	0	6.93	4.59E-04
CG8031	CG8031	17.64	0	6.93	4.60E-04
CG6121	Tip60	17.21	0	6.90	5.14E-04
CG9865	CG9865	17.13	0	6.89	5.25E-04
CG33199	CG33199	16.51	0	6.84	6.16E-04
CG42489	CG42489	16.18	0	6.81	6.76E-04
CG5758	CG5758	15.7	0	6.77	7.70E-04
CG4186	CG4186	15.36	0	6.74	8.46E-04
CG32207	CG32207	16420.68	111.11	6.73	1.72E-13
CG3428	pall	14.88	0	6.69	9.68E-04
CG11601	CG11601	14.8	0	6.68	9.90E-04
CG41267	CG41267	14.71	0	6.67	1.02E-03
CG32561	xmas-1	14.59	0	6.66	1.05E-03
CG6951	CG6951	14.48	0	6.65	1.08E-03
CG18374	Gyk	14.11	0	6.61	1.21E-03
CG14117	CG14117	14.11	0	6.61	1.21E-03
CG12534	CG12534	14.1	0	6.61	1.21E-03
CG5783	CG5783	14.09	0	6.61	1.21E-03
CG42350	CG42350	14.07	0	6.61	1.22E-03
CG8374	dmt	13.96	0	6.60	1.26E-03
CG11041	CG11041	13.91	0	6.59	1.28E-03
CG1463	CG1463	13.87	0	6.59	1.29E-03
CG7137	CG7137	13.71	0	6.57	1.36E-03
CG13038	CG13038	13.69	0	6.57	1.37E-03
CG4537	CG4537	13.56	0	6.56	1.42E-03
CG15352	CG15352	13.46	0	6.55	1.46E-03
CG11723	CG11723	13.45	0	6.55	1.47E-03
CG11447	CG11447	13.45	0	6.55	1.47E-03
CG2118	CG2118	13.38	0	6.54	1.50E-03
CG13329	cid	13.25	0	6.52	1.56E-03
CG13626	Syx18	13.23	0	6.52	1.57E-03
CG34446	CG34446	13.13	0	6.51	1.61E-03
CG11261	CG11261	13.01	0	6.50	1.67E-03
CG31457	CG31457	12.88	0	6.48	1.74E-03
CG32442	CG32442	12.84	0	6.48	1.76E-03
CG12783	CG12783	24.46	0.1	6.43	1.57E-04
CG5618	CG5618	11.88	0	6.37	2.38E-03
CG1397	CG1397	11.76	0	6.35	2.47E-03
CG30286	CG30286	11.72	0	6.35	2.50E-03
CG32298	CG32298	11.51	0	6.32	2.68E-03
CG42254	CG42254	11.02	0	6.26	3.14E-03
CG1363	blow	10.97	0	6.26	3.19E-03
CG14578	CG14578	10.73	0	6.22	3.46E-03
CG14882	CG14882	10.71	0	6.22	3.48E-03
CG34002	CG34002	10.7	0	6.22	3.49E-03
CG6459	CG6459	2185.26	21.04	6.21	9.82E-12
CG17982	CG17982	10.53	0	6.20	3.70E-03
CG11200	CG11200	10.49	0	6.19	3.75E-03
CG7864	CG7864	10.49	0	6.19	3.75E-03
CG33111	CG33111	10.31	0	6.17	3.99E-03
CG4694	her	10.1	0	6.14	4.28E-03
CG7234	GluRIIB	18.81	0.09	6.13	5.43E-04
CG9256	Nhe2	9.95	0	6.12	4.51E-03
CG6224	dbo	9.83	0	6.10	4.70E-03
CG9335	CG9335	9.34	0	6.03	5.59E-03
CG34302	CG34302	9.33	0	6.03	5.61E-03
CG10067	Act57B	21.7	0.14	6.00	3.33E-04
CG15390	CG15390	9.07	0	5.99	6.16E-03
CG31622	Gr39a	8.81	0	5.94	6.77E-03
CG31957	CG31957	8.56	0	5.90	7.42E-03
CG12236	CG12236	8.45	0	5.88	7.73E-03
CG17970	Cyp4ac2	8.36	0	5.87	8.00E-03
CG12346	cag	8.18	0	5.84	8.56E-03
CG1401	cul-5	8.12	0	5.83	8.75E-03
CG15362	CG15362	8.05	0	5.82	8.99E-03
CG4733	CG4733	8.01	0	5.81	9.13E-03
CG13919	CG13919	7.73	0	5.76	1.02E-02
CG13472	CG13472	7.68	0	5.75	1.04E-02
CG11186	toy	7.66	0	5.75	1.04E-02
CG2711	dwg	7.48	0	5.71	1.12E-02
CG14450	CG14450	7.32	0	5.68	1.19E-02
CG15032	CG15032	7.27	0	5.67	1.22E-02
CG11655	CG11655	7.27	0	5.67	1.22E-02
CG14757	CG14757	7.25	0	5.67	1.23E-02
CG7101	CG7101	7.12	0	5.64	1.29E-02
CG7466	CG7466	6.75	0	5.57	1.50E-02
CG33196	dp	11.95	0.08	5.56	3.49E-03
CG10344	CG10344	6.56	0	5.53	1.62E-02
CG7062	Rab-RP3	6.46	0	5.50	1.69E-02
CG34240	CG34240	6.36	0	5.48	1.77E-02
CG33324	CG33324	6.28	0	5.47	1.83E-02
CG32554	CG32554	6.27	0	5.46	1.83E-02
CG2292	CG2292	6.17	0	5.44	1.91E-02
CG10209	CG10209	6.17	0	5.44	1.91E-02
CG5109	Pcl	6.17	0	5.44	1.91E-02
CG6043	CG6043	6.11	0	5.43	1.96E-02
CG11219	PIP82	7.9	0.03	5.43	1.14E-02
CG6900	CG6900	6.06	0	5.41	2.01E-02
CG31122	CG31122	5.96	0	5.39	2.09E-02
CG14232	CG14232	5.94	0	5.39	2.11E-02
CG1360	CG1360	74.53	1.18	5.38	3.10E-06
CG32132	CG32132	5.79	0	5.35	2.25E-02
CG13964	CG13964	5.77	0	5.35	2.27E-02
CG33985	CG33985	5.65	0	5.32	2.40E-02
CG9059	CG9059	5.63	0	5.31	2.42E-02
CG30055	CG30055	5.62	0	5.31	2.43E-02
CG18539	CG18539	5.62	0	5.31	2.43E-02
CG1747	Sk1	5.52	0	5.28	2.54E-02
CG8213	CG8213	5.95	0.01	5.26	2.26E-02
CG7005	Esp	25.09	0.4	5.17	3.57E-04
CG6254	CG6254	5.01	0	5.15	3.19E-02
CG17957	Sry-alpha	4.98	0	5.14	3.24E-02
CG33755	gdl-ORF39	4.82	0	5.09	3.49E-02
CG32356	ImpE1	4.72	0	5.06	3.66E-02
CG32204	CG32204	7.88	0.07	5.05	1.37E-02
CG30038	CG30038	4.59	0	5.02	3.89E-02
CG31361	dpr17	25.83	0.47	5.02	3.69E-04
CG9834	endoB	4.57	0	5.02	3.92E-02
CG6052	CG6052	4.53	0	5.01	4.00E-02
CG9692	CG9692	6.31	0.04	5.00	2.26E-02
CG12508	CG12508	4.45	0	4.98	4.16E-02
CG2657	CG2657	11.47	0.16	4.98	5.41E-03
CG12952	sage	4.35	0	4.95	4.36E-02
CG13617	CG13617	4.26	0	4.92	4.55E-02
CG11459	CG11459	4.21	0	4.90	4.67E-02
CG41327	CG41327	4.17	0	4.89	4.76E-02
CG34231	CG34231	4.11	0	4.87	4.90E-02
CG11101	pwn	4.51	0.01	4.87	4.33E-02
CG41336	CG41336	4.1	0	4.87	4.93E-02
CG12172	Spn43Aa	8.12	0.14	4.60	1.60E-02
CG17121	CG17121	34.82	1.09	4.39	2.77E-04
CG33126	NLaz	20.96	0.8	4.07	2.08E-03
CG15364	CG15364	44.98	1.84	4.06	2.16E-04
CG33521	CG33521	80.03	3.6	3.96	5.25E-05
CG13422	CG13422	13.77	0.56	3.91	7.52E-03
CG5161	CG5161	194.27	9.38	3.88	9.59E-06
CG6416	CG6416	6.08	0.2	3.88	4.26E-02
CG33484	zormin	8.3	0.31	3.87	2.40E-02
CG9020	Aats-arg	78.02	3.94	3.80	8.61E-05
CG15879	CG15879	39.43	2	3.76	5.60E-04
CG32773	CG32773	11.48	0.53	3.72	1.37E-02
CG17706	CG40282	13.73	0.72	3.60	1.04E-02
CG6231	CG6231	14.3	0.78	3.55	9.92E-03
CG1265	CG1265	34.89	2.32	3.38	1.59E-03
CG11660	CG11660	21.97	1.44	3.36	4.75E-03
CG2006	CG2006	553.98	38.9	3.35	2.78E-05
CG9032	sun	2942.49	207.67	3.35	1.76E-05
CG10997	Clic	481.52	35.3	3.29	3.98E-05
CG4676	CG4676	17.29	1.3	3.16	1.07E-02
CG13139	CG13139	40.75	3.2	3.15	1.79E-03
CG1112	alpha-Est7	14.82	1.12	3.13	1.50E-02
CG6502	E(z)	14.65	1.18	3.05	1.71E-02
CG8405	CG8405	85.75	7.43	3.03	6.32E-04
CG42307	CG42307	28.09	2.41	3.01	5.05E-03
CG9921	CG9921	233.44	20.99	2.99	2.27E-04
CG7215	CG7215	77.63	7.1	2.96	9.30E-04
CG17033	CG17033	16.5	1.44	2.95	1.55E-02
CG7224	CG7224	1291.15	120.18	2.95	1.30E-04
CG13895	CG13895	12.55	1.08	2.95	2.56E-02
CG2221	l(1)G0289	35.61	3.24	2.94	3.68E-03
CG11926	CG11926	26.15	2.36	2.94	6.64E-03
CG12050	CG12050	39.69	3.66	2.93	3.11E-03
CG30185	CG30185	64.03	5.99	2.92	1.39E-03
CG34031	CG34031	43.94	4.37	2.82	3.30E-03
CG9743	CG9743	39.69	3.95	2.82	3.96E-03
CG2099	RpL35A	825.53	83.95	2.82	2.53E-04
CG32850	CG32850	64.02	6.43	2.82	1.84E-03
CG9131	slmo	24.6	2.46	2.79	9.64E-03
CG7762	Rpn1	37.39	3.85	2.77	4.90E-03
CG7637	CG7637	605.21	65.96	2.72	4.24E-04
CG40127	CG40127	461.16	53.46	2.63	6.71E-04
CG15653	CG15653	40.87	4.79	2.59	6.42E-03
CG7823	RhoGDI	240.25	28.85	2.58	1.10E-03
CG1983	CG1983	16.41	2	2.50	3.01E-02
CG34335	CG34335	317295.75	40848.33	2.48	8.95E-04
CG6759	cdc16	19.53	2.47	2.46	2.49E-02
CG40411	Parp	151.06	19.84	2.45	2.37E-03
CG3353	CG3353	51.62	6.75	2.44	6.79E-03
CG2976	CG2976	62.73	8.28	2.43	5.55E-03
CG8922	RpS5a	4808.79	647.21	2.42	1.22E-03
CG7362	CG7362	28.02	3.77	2.39	1.71E-02
CG6593	Pp1alpha-96A	33.68	4.57	2.38	1.36E-02
CG2928	Reg-5	30.26	4.13	2.37	1.60E-02
CG17667	CG17667	14.23	1.97	2.32	4.78E-02
CG33748	primo-1	791.73	114.29	2.31	2.12E-03
CG34132	CG34132	158.4	22.84	2.31	3.66E-03
CG8660	Fibp	19.86	2.8	2.31	3.13E-02
CG31676	CG31676	463.55	71.6	2.22	3.44E-03
CG5687	CG5687	111.57	17.41	2.20	6.68E-03
CG1071	E2f2	39.28	6.08	2.20	1.70E-02
CG31229	CG31229	56.36	8.87	2.18	1.23E-02
CG3792	CG3792	56.14	8.9	2.17	1.27E-02
CG32523	CG32523	37.75	5.96	2.17	1.90E-02
CG12400	CG12400	342.24	57.65	2.09	5.90E-03
CG17691	CG17691	66.35	11.55	2.04	1.57E-02
CG32686	CG32686	42.09	7.37	2.02	2.39E-02
CG14683	CG14683	36.31	6.35	2.02	2.75E-02
CG10674	CG10674	101.84	18.01	2.02	1.24E-02
CG10512	CG10512	100.15	17.73	2.01	1.26E-02
CG2219	CG2219	124.29	22.31	2.00	1.18E-02
CG9849	CG9849	27.12	4.82	1.99	3.90E-02
CG5510	CG5510	70.52	12.79	1.98	1.76E-02
CG9521	CG9521	28.39	5.28	1.93	4.23E-02
CG33785	CG33785	37.45	7.05	1.92	3.38E-02
CG11027	Arf102F	380.45	72.92	1.91	1.12E-02
CG18135	CG18135	58.05	11.16	1.89	2.54E-02
CG13663	CG13663	31.83	6.11	1.89	4.17E-02
CG9796	CG9796	193.63	37.74	1.88	1.44E-02
CG10640	Uev1A	278.92	54.68	1.87	1.34E-02
CG5706	CG5706	57.02	11.52	1.82	3.08E-02
CG8053	eIF-1A	287.11	58.31	1.82	1.57E-02
CG4673	CG4673	82.49	16.78	1.81	2.52E-02
CG3937	cher	40.85	8.28	1.81	3.99E-02
CG17489	RpL5	2324.61	481.67	1.79	1.42E-02
CG7085	l(2)s5379	42.25	8.85	1.77	4.34E-02
CG8361	HLHm7	47.77	10.02	1.77	3.99E-02
CG9688	mRpS18C	38.49	8.1	1.76	4.72E-02
CG15717	CG15717	43.09	9.16	1.75	4.49E-02
CG10849	Sc2	97.89	20.91	1.75	2.82E-02
CG11885	CG11885	76.28	16.31	1.74	3.20E-02
CG34163	CG34163	95.25	20.96	1.70	3.22E-02
CG40049	mRpS5	93.86	21.06	1.67	3.50E-02
CG30109	CG30109	64.45	14.72	1.65	4.49E-02
CG15019	CG15019	5330.17	1227.26	1.64	2.39E-02
CG12372	spt4	72.02	16.82	1.62	4.62E-02
CG7872	CG7872	107.5	25.4	1.60	4.09E-02
CG2803	Tina-1	110.45	26.28	1.59	4.16E-02
CG14029	vri	172.73	41.14	1.59	3.66E-02
CG15162	MESR3	215.97	52.48	1.56	3.80E-02
CG33002	mRpL27	119.85	29.15	1.56	4.43E-02
CG32031	Argk	580.29	143.87	1.53	3.66E-02
CG1787	Hexo2	253.56	62.99	1.53	4.07E-02
CG10071	RpL29	14352.71	3619.01	1.51	3.67E-02
CG1524	RpS14a	1106.69	281.93	1.50	4.00E-02
CG3949	hoip	410.96	107.49	1.46	4.79E-02
CG4863	RpL3	2586	689.19	1.43	4.80E-02
CG6846	RpL26	3629.7	971.42	1.43	4.87E-02

OSC < Δmbt-OSC

ID	Symbol	OSC-FPKM	Δmbt-OSC FPKM	OSC/Δmbt-OSC FC	PValue
CG8486	CG8486	2.41	274.62	-7.22	9.13E-12
CG30283	CG30283	0	130.49	-10.29	1.27E-11
CG7660	pxt	0	73.44	-9.46	1.17E-09
CG2233	CG2233	0	70.3	-9.40	1.64E-09
CG31997	CG31997	1.47	112.45	-6.60	2.48E-09
CG4193	dhd	0	65.1	-9.28	2.94E-09
CG6081	Cyp28d2	0	59.34	-9.15	5.92E-09
CG10125	zpg	0.08	59.59	-8.53	8.99E-09
CG7615	fig	2.64	119.6	-5.90	9.72E-09
CG30380	CG30380	0.58	72.3	-7.12	1.09E-08
CG14932	CG14932	1.53	93.75	-6.28	1.14E-08
CG9925	CG9925	0.03	46.27	-8.52	4.56E-08
CG12019	Cdc37	0	44.63	-8.74	4.87E-08
CG11985	CG11985	0	44.61	-8.74	4.88E-08
CG33481	dpr7	0.68	60.09	-6.66	5.66E-08
CG32681	CG32681	3.83	103.76	-5.18	1.23E-07
CG12086	cue	0	35.96	-8.43	2.27E-07
CG18505	Acyp2	0	35.21	-8.40	2.63E-07
CG11828	CG11828	0.91	51.33	-6.08	3.07E-07
CG33926	CG33926	0.23	37.7	-7.13	4.55E-07
CG13922	mRpL46	0	31.8	-8.25	5.32E-07
CG4114	ex	0	30.95	-8.21	6.40E-07
CG8936	Arpc3B	0.37	38	-6.68	6.74E-07
CG40968	CG40968	0	30.07	-8.17	7.78E-07
CG15930	CG15930	0.08	31	-7.58	9.84E-07
CG13741	CG13741	0	28.77	-8.11	1.05E-06
CG3506	vas	0.57	37.22	-6.18	1.35E-06
CG1542	CG1542	0	27.69	-8.05	1.35E-06
CG9514	CG9514	0	27.13	-8.03	1.55E-06
CG31717	CG31717	0	26.64	-8.00	1.75E-06
CG41326	CG41326	0	26.46	-7.99	1.83E-06
CG14193	CG14193	5.61	87.27	-4.40	2.35E-06
CG6927	CG6927	0	25.25	-7.92	2.48E-06
CG15009	ImpL2	1.43	43.35	-5.26	2.80E-06
CG33468	CG33468	0	24.36	-7.87	3.13E-06
CG31115	CG31115	0	24.02	-7.85	3.43E-06
CG10109	L	0	23.99	-7.85	3.45E-06
CG40609	CG40609	0	23.21	-7.80	4.27E-06
CG1119	Gnf1	0	22.58	-7.76	5.08E-06
CG9361	Task7	0.2	25.87	-6.71	5.24E-06
CG34339	CG34339	0	22.44	-7.75	5.28E-06
CG34358	shakB	0	22.36	-7.75	5.40E-06
CG8589	Tejas	0.08	23.84	-7.21	5.56E-06
CG40300	AGO3	2.14	45.96	-4.81	5.69E-06
CG1629	yellow-h	3.08	53.53	-4.53	6.57E-06
CG7997	CG7997	0	21.5	-7.69	6.91E-06
CG40498	CG40498	0	21.22	-7.67	7.50E-06
CG14341	CG14341	0	21.21	-7.67	7.52E-06
CG9475	Rpt3R	0	21.14	-7.67	7.68E-06
CG30484	CG30484	0.39	25.69	-6.07	9.93E-06
CG32368	CG32368	24.59	216.07	-3.60	1.17E-05
CG8722	Nup44A	0	19.48	-7.55	1.27E-05
CG11094	dsx	12.85	121.71	-3.71	1.49E-05
CG41520	CG41520	1.67	35.31	-4.76	1.68E-05
CG6967	CG6967	0.21	21.57	-6.41	1.72E-05
CG10308	CycJ	0	18.41	-7.47	1.78E-05
CG14036	CG14036	0	17.67	-7.41	2.27E-05
CG5637	nos	0	17.66	-7.41	2.28E-05
CG3979	Indy	14.48	123.9	-3.56	2.56E-05
CG7865	PNGase	0	16.27	-7.29	3.67E-05
CG5232	Sas	0	16.1	-7.28	3.90E-05
CG30170	bgcn	0.7	23.33	-5.27	3.97E-05
CG30016	CG30016	0	15.96	-7.26	4.10E-05
CG9662	CG9662	0	15.86	-7.25	4.24E-05
CG31191	CG31191	0.01	15.65	-7.14	4.94E-05
CG42316	RhoGAP102A	0.14	17.2	-6.40	5.19E-05
CG10192	ofs	0	15.25	-7.20	5.30E-05
CG32700	CG32700	0.04	15.52	-6.87	6.11E-05
CG1740	Ntf-2	18.23	131.56	-3.32	6.22E-05
CG31534	CG31534	0	14.33	-7.11	7.48E-05
CG34290	CG34290	0	14.32	-7.11	7.51E-05
CG14327	CG14327	0	14.24	-7.10	7.74E-05
CG40467	CG40467	0.6	20.03	-5.23	7.94E-05
CG34355	CG34355	0.06	15	-6.68	8.14E-05
CG11052	CG11052	0.41	18.36	-5.53	8.23E-05
CG6185	CG6185	0.2	16	-6.02	9.70E-05
CG42230	bbg	4.21	42.53	-3.77	9.72E-05
CG14643	TwdlG	0	13.54	-7.03	1.02E-04
CG8776	CG8776	3.35	36.48	-3.86	1.13E-04
CG12698	CG12698	0.05	13.91	-6.64	1.17E-04
CG6490	CG6490	4.37	42.12	-3.70	1.19E-04
CG32538	gfA	0	13.04	-6.97	1.24E-04
CG10445	CG10445	0	12.85	-6.95	1.34E-04
CG4977	kek2	0	12.69	-6.94	1.43E-04
CG14905	CG14905	0	12.65	-6.93	1.46E-04
CG7000	Snmp	0.03	13.03	-6.70	1.51E-04
CG8344	RpIII128	0	12.55	-6.92	1.52E-04
CG1507	Pur-alpha	0	12.48	-6.91	1.57E-04
CG12367	Pimet	0	12.46	-6.91	1.58E-04
CG32476	mthl14	0	12.42	-6.90	1.61E-04
CG32817	CG32817	0	12.41	-6.90	1.61E-04
CG8497	Rhp	0	12.21	-6.88	1.75E-04
CG42272	CG42272	0	12.18	-6.88	1.78E-04
CG7166	CG7166	0.14	13.68	-6.07	1.86E-04
CG5060	CG5060	3.3	33.29	-3.75	1.90E-04
CG17216	KP78b	0	11.94	-6.85	1.97E-04
CG1693	tty	0.15	13.43	-5.99	2.12E-04
CG2004	CG2004	0	11.61	-6.81	2.27E-04
CG2381	Syt7	0.33	14.72	-5.44	2.29E-04
CG7259	Best4	0.12	12.91	-6.09	2.34E-04
CG3509	CG3509	0.04	12.02	-6.51	2.41E-04
CG33927	CG33927	0	11.41	-6.78	2.47E-04
CG5992	Adgf-A	0.13	12.65	-6.01	2.70E-04
CG4249	c(2)M	0	11.16	-6.75	2.76E-04
CG14810	CG14810	0.11	12.38	-6.09	2.80E-04
CG42263	CG42263	0	11.03	-6.73	2.93E-04
CG8023	eIF4E-3	0.13	12.37	-5.98	3.03E-04
CG7777	CG7777	0.1	11.95	-6.09	3.23E-04
CG12194	CG12194	0	10.75	-6.70	3.32E-04
CG14516	CG14516	0	10.74	-6.70	3.34E-04
CG4247	mRpS10	4.03	33.89	-3.50	3.40E-04
CG14948	dpr18	0.11	11.91	-6.03	3.41E-04
CG7512	CG7512	0.43	14.36	-5.13	3.41E-04
CG16979	CG16979	0	10.68	-6.69	3.43E-04
CG30325	CG30325	0.02	10.96	-6.54	3.45E-04
CG9380	CG9380	0.47	14.52	-5.05	3.56E-04
CG7590	scyl	32.02	164	-2.83	3.72E-04
CG3767	Jhl-26	9.8	60.86	-3.09	3.74E-04
CG10738	CG10738	1.98	22.86	-3.91	3.89E-04
CG32568	CG32568	0	10.4	-6.65	3.90E-04
CG2380	Nfl	0.01	10.43	-6.56	4.15E-04
CG11822	nAcRbeta-21C	0	10.25	-6.63	4.19E-04
CG4960	CG4960	0.37	13.37	-5.18	4.25E-04
CG6588	Fas1	26.24	134.22	-2.82	4.29E-04
CG4790	fs(1)M3	0.01	10.35	-6.55	4.30E-04
CG9668	Rh4	0	10.15	-6.62	4.39E-04
CG18522	CG18522	3.3	28.81	-3.55	4.52E-04
CG13373	CG13373	0	10.04	-6.60	4.62E-04
CG32483	CG32483	4.07	32.32	-3.42	4.68E-04
CG13694	CG13694	0	9.95	-6.59	4.82E-04
CG34288	CG34288	0	9.95	-6.59	4.82E-04
CG6859	CG6859	96.51	441.7	-2.67	4.91E-04
CG32217	Su(Tpl)	0	9.83	-6.57	5.11E-04
CG4928	CG4928	0.9	16.08	-4.43	5.16E-04
CG33679	CG33679	0.43	13.17	-5.00	5.37E-04
CG8262	ana2	0	9.58	-6.53	5.76E-04
CG18810	CG18810	0	9.54	-6.53	5.87E-04
CG8121	CG8121	0	9.48	-6.52	6.05E-04
CG3831	CG3831	0	9.45	-6.51	6.14E-04
CG9643	CG9643	0	9.44	-6.51	6.17E-04
CG14400	CG14400	0	9.44	-6.51	6.17E-04
CG5370	Dcp-1	0	9.41	-6.51	6.26E-04
CG5569	CG5569	0	9.4	-6.51	6.29E-04
CG10965	Corp	1.18	16.98	-4.16	6.31E-04
CG11560	CG11560	0	9.39	-6.50	6.32E-04
CG14799	CG14799	0.31	11.94	-5.20	6.42E-04
CG40239	CG40239	0	9.3	-6.49	6.60E-04
CG1503	CG1503	1.08	16.3	-4.22	6.63E-04
CG9509	CG9509	0	9.29	-6.49	6.64E-04
CG9706	CG9706	0	9.28	-6.49	6.67E-04
CG4894	Ca-alpha1D	0.01	9.41	-6.41	6.73E-04
CG11155	CG11155	2.48	23.09	-3.62	6.78E-04
CG5878	alpha4GT2	0	9.21	-6.48	6.90E-04
CG14964	CG14964	1.62	18.9	-3.90	6.92E-04
CG9874	Tbp	0	9.19	-6.47	6.97E-04
CG3981	Unc-76	0	9.08	-6.46	7.37E-04
CG32625	CG32625	14.14	72.41	-2.82	7.56E-04
CG11760	CG11760	0	8.96	-6.44	7.82E-04
CG15151	rdo	7.48	44.18	-3.01	7.84E-04
CG9902	CG9902	25.86	119.49	-2.68	8.19E-04
CG4859	Mmp1	0	8.78	-6.41	8.57E-04
CG8789	wnd	2.42	21.85	-3.57	8.61E-04
CG6584	SelR	0	8.72	-6.40	8.83E-04
CG10859	CG10859	2.56	22.25	-3.52	9.12E-04
CG16749	CG16749	0	8.61	-6.38	9.34E-04
CG4559	Idgf3	1.73	18.27	-3.77	9.69E-04
CG3027	pyd3	1.96	19.31	-3.68	9.73E-04
CG9470	MtnA	62.91	262.75	-2.54	9.81E-04
CG18578	Ugt86Da	3.55	26.22	-3.31	9.85E-04
CG17604	c(3)G	2.68	22.44	-3.47	9.92E-04
CG4211	nonA	0	8.47	-6.36	1.00E-03
CG3743	MTF-1	0	8.46	-6.36	1.01E-03
CG2913	yin	4.54	30.14	-3.17	1.03E-03
CG30296	CG30296	0.05	8.93	-6.01	1.05E-03
CG3879	Mdr49	0.32	10.76	-5.02	1.10E-03
CG12283	kek1	25.4	111.49	-2.60	1.13E-03
CG34259	CG34259	0.17	9.66	-5.43	1.16E-03
CG40305	FucTC	0	8.16	-6.30	1.18E-03
CG32711	CG32711	0	8.14	-6.30	1.19E-03
CG4269	CG4269	2.79	22.08	-3.39	1.22E-03
CG8526	CG8526	0	8.02	-6.28	1.27E-03
CG10962	CG10962	6.58	36.97	-2.94	1.31E-03
CG4302	CG4302	0	7.85	-6.25	1.39E-03
CG13841	CG13841	3.65	25.02	-3.20	1.40E-03
CG32778	CG32778	0	7.84	-6.25	1.40E-03
CG9068	CG9068	0	7.82	-6.24	1.42E-03
CG13907	CG13907	9.81	48.7	-2.77	1.44E-03
CG1674	CG1674	6.75	36.71	-2.89	1.51E-03
CG32436	CG32436	0.94	13.24	-4.10	1.51E-03
CG17905	ChLD3	0	7.69	-6.22	1.52E-03
CG17769	And	0	7.68	-6.22	1.53E-03
CG33494	CG33494	1.03	13.47	-4.01	1.62E-03
CG42401	mun	0.01	7.69	-6.12	1.63E-03
CG2987	alpha-catenin-related	0.02	7.77	-6.05	1.66E-03
CG33257	CG33257	0.07	8.17	-5.74	1.69E-03
CG9935	CG9935	1.65	16.01	-3.64	1.73E-03
CG6215	CkIIalpha-i1	0	7.41	-6.17	1.77E-03
CG14931	CG14931	0.08	8.16	-5.67	1.77E-03
CG13252	CG13252	10.04	47.68	-2.71	1.80E-03
CG8779	nrm	4.76	28.07	-3.00	1.82E-03
CG2999	unc-13	31.12	122.95	-2.45	1.87E-03
CG5381	CG5381	0	7.31	-6.15	1.87E-03
CG2670	Taf7	0	7.28	-6.14	1.90E-03
CG4698	Wnt4	0.13	8.31	-5.41	1.98E-03
CG31163	CG31163	18.89	78.41	-2.52	1.98E-03
CG32577	disco-r	0	7.2	-6.13	1.99E-03
CG13512	CG13512	2.14	17.53	-3.42	2.02E-03
CG1435	CBP	0.71	11.4	-4.23	2.03E-03
CG8468	CG8468	0	7.15	-6.12	2.05E-03
CG33680	CG33680	0.09	7.9	-5.56	2.11E-03
CG12918	CG12918	0	6.98	-6.08	2.25E-03
CG6414	CG6414	0	6.95	-6.08	2.29E-03
CG2665	PebII	0	6.91	-6.07	2.34E-03
CG14321	CG14321	2.93	20.03	-3.19	2.35E-03
CG3408	CG3408	0	6.89	-6.06	2.37E-03
CG9504	Eo	0.33	9.12	-4.75	2.40E-03
CG4841	CG4841	0	6.81	-6.05	2.48E-03
CG11714	CG11714	0	6.79	-6.04	2.51E-03
CG18812	CG18812	2.43	17.9	-3.28	2.51E-03
CG34343	sick	1.41	13.82	-3.64	2.58E-03
CG31147	mthl11	0.21	8.29	-5.04	2.59E-03
CG18166	CG18166	0	6.7	-6.02	2.65E-03
CG5738	lolal	4.16	24.03	-2.96	2.66E-03
CG5955	CG5955	0	6.62	-6.01	2.77E-03
CG7476	mthl7	0	6.58	-6.00	2.84E-03
CG7628	CG7628	5.11	27.02	-2.84	2.86E-03
CG40119	CG40119	0	6.56	-5.99	2.87E-03
CG32706	CG32706	6.66	32.09	-2.72	3.00E-03
CG3717	bcn92	0	6.48	-5.98	3.01E-03
CG31718	CG31718	0.24	8.16	-4.90	3.02E-03
CG34378	Pvf3	1.13	12.23	-3.75	3.04E-03
CG4484	CG4484	1.78	14.78	-3.43	3.06E-03
CG32006	CG32006	0	6.45	-5.97	3.06E-03
CG9154	CG9154	0	6.43	-5.97	3.10E-03
CG12161	CG12161	1.1	12.03	-3.76	3.14E-03
CG10996	CG10996	0	6.4	-5.96	3.15E-03
CG7375	CG7375	6.8	32.03	-2.69	3.29E-03
CG15531	CG15531	0.82	10.69	-3.96	3.36E-03
CG8870	CG8870	0	6.25	-5.93	3.45E-03
CG9441	Pu	6.38	30.28	-2.70	3.49E-03
CG34416	Ank2	0.19	7.6	-5.00	3.49E-03
CG6817	foi	35.89	125.43	-2.28	3.52E-03
CG17816	CG17816	1.36	12.71	-3.57	3.59E-03
CG40006	CG40006	4.49	23.72	-2.84	3.65E-03
CG32557	CG32557	0	6.15	-5.90	3.66E-03
CG8095	scb	3.01	18.61	-3.04	3.70E-03
CG10391	Cyp310a1	1.39	12.74	-3.54	3.70E-03
CG34366	CG34366	1.91	14.68	-3.32	3.71E-03
CG15332	CG15332	1.16	11.82	-3.67	3.74E-03
CG9449	CG9449	0.76	10.18	-3.98	3.75E-03
CG10998	r-cup	0	6.09	-5.89	3.80E-03
CG15450	CG15450	0	6.08	-5.89	3.82E-03
CG31674	CG31674	11.89	47.51	-2.46	3.84E-03
CG33140	CG33140	0	6.03	-5.87	3.94E-03
CG8557	CG8557	4.31	22.75	-2.84	3.96E-03
CG7533	chrb	21.2	76.56	-2.32	4.00E-03
CG42357	CG42357	0	5.99	-5.87	4.04E-03
CG9476	alphaTub85E	0.04	6.36	-5.60	4.06E-03
CG6776	CG6776	22.46	80.15	-2.30	4.10E-03
CG11838	Oseg3	1.39	12.43	-3.51	4.15E-03
CG10240	Cyp6a22	0	5.91	-5.85	4.24E-03
CG8596	CG8596	0	5.88	-5.84	4.32E-03
CG32274	Drs-l	0	5.82	-5.82	4.48E-03
CG5083	Rbf2	0	5.79	-5.82	4.56E-03
CG12630	tio	0.54	8.79	-4.17	4.58E-03
CG2893	CG2893	0.05	6.22	-5.49	4.62E-03
CG12970	CG12970	0.04	6.14	-5.55	4.63E-03
CG13775	CG13775	0	5.71	-5.80	4.80E-03
CG15822	CG15822	0.04	6.08	-5.54	4.80E-03
CG17684	CG17684	8.24	34.3	-2.51	4.80E-03
CG34258	CG34258	0	5.7	-5.80	4.83E-03
CG40160	CG40160	2.67	16.45	-3.03	4.89E-03
CG30110	CG30110	0.03	5.97	-5.59	4.89E-03
CG18193	CG18193	0.33	7.67	-4.51	4.96E-03
CG7676	cona	63.21	199.68	-2.13	5.06E-03
CG41473	CG41473	0	5.6	-5.77	5.14E-03
CG11411	fs(1)N	1.76	13.16	-3.28	5.18E-03
CG6058	Ald	23.88	80.93	-2.23	5.23E-03
CG15580	CG15580	0	5.56	-5.76	5.27E-03
CG3556	CG3556	2.65	16.1	-3.01	5.32E-03
CG31000	heph	8.25	33.63	-2.48	5.35E-03
CG5455	CG5455	0	5.52	-5.75	5.40E-03
CG31145	CG31145	1.51	12.16	-3.37	5.40E-03
CG3105	CG3105	0.87	9.69	-3.75	5.65E-03
CG12505	Arc1	375.64	1112.27	-2.04	5.72E-03
CG13611	CG13611	0.41	7.76	-4.30	5.74E-03
CG14354	CG14354	0	5.41	-5.72	5.79E-03
CG31141	CG31141	0	5.4	-5.72	5.83E-03
CG3429	swa	0.07	5.96	-5.29	5.87E-03
CG4723	CG4723	0	5.36	-5.71	5.98E-03
CG8403	SP2353	1.68	12.46	-3.26	6.02E-03
CG3340	Kr	0.02	5.55	-5.57	6.03E-03
CG18467	CG18467	0	5.33	-5.70	6.10E-03
CG8620	CG8620	2.53	15.22	-2.99	6.16E-03
CG6790	CG6790	0	5.27	-5.68	6.34E-03
CG3308	CG3308	0	5.26	-5.68	6.38E-03
CG14073	CG14073	6.78	28.04	-2.50	6.49E-03
CG9908	disco	0	5.23	-5.67	6.50E-03
CG6687	CG6687	0.07	5.78	-5.25	6.55E-03
CG10808	synaptogyrin	2.26	14.14	-3.04	6.57E-03
CG11060	CG11060	0	5.21	-5.67	6.59E-03
CG42283	5PtaseI	0	5.21	-5.67	6.59E-03
CG17594	scro	0.13	6.13	-4.98	6.59E-03
CG6494	h	1.26	10.76	-3.43	6.60E-03
CG10034	tj	30.39	95.97	-2.13	6.69E-03
CG5080	CG5080	0.61	8.25	-3.94	6.86E-03
CG33056	CG33056	0	5.14	-5.65	6.89E-03
CG41476	CG41476	0	5.14	-5.65	6.89E-03
CG41026	CG41026	0	5.13	-5.65	6.94E-03
CG11409	CG11409	0.15	6.15	-4.88	6.96E-03
CG7052	TepII	0.59	8.13	-3.96	7.00E-03
CG31021	CG31021	0.11	5.88	-5.03	7.15E-03
CG32433	Gr77a	0	5.07	-5.63	7.21E-03
CG33970	CG33970	0.26	6.64	-4.53	7.23E-03
CG4015	Fcp3C	0	5.06	-5.63	7.26E-03
CG34432	CG34432	1.09	9.92	-3.50	7.28E-03
CG6137	aub	1.2	10.3	-3.43	7.30E-03
CG40298	CG40298	0.15	6.05	-4.86	7.38E-03
CG5731	CG5731	0.13	5.94	-4.93	7.39E-03
CG30390	CG30390	0	5.02	-5.62	7.45E-03
CG34103	BG642167	0	5.01	-5.61	7.50E-03
CG30193	CG30193	0	5	-5.61	7.55E-03
CG13962	CG13962	2.51	14.47	-2.93	7.62E-03
CG42242	beat-VII	0	4.98	-5.60	7.65E-03
CG33198	pen-2	12.3	42.86	-2.26	7.65E-03
CG8046	CG8046	0.6	7.95	-3.91	7.80E-03
CG4472	Idgf1	5.09	22.09	-2.56	7.87E-03
CG8958	CG8958	0	4.9	-5.58	8.07E-03
CG15684	CG15684	0	4.89	-5.58	8.12E-03
CG13320	Sans	0.09	5.55	-5.06	8.15E-03
CG13766	CG13766	0	4.87	-5.57	8.23E-03
CG31015	PH4alphaPV	1.02	9.28	-3.49	8.64E-03
CG14864	CG14864	0.03	5.06	-5.36	8.68E-03
CG4962	CG4962	5.88	23.88	-2.47	8.71E-03
CG4986	Mst57Dc	1.39	10.51	-3.27	8.72E-03
CG30440	CG30440	9.69	34.54	-2.29	8.74E-03
CG4613	CG4613	1.5	10.86	-3.21	8.76E-03
CG10993	CG10993	0	4.77	-5.54	8.80E-03
CG33775	CG33775	0	4.75	-5.54	8.92E-03
CG14962	CG14962	0	4.75	-5.54	8.92E-03
CG41339	CG41339	0.1	5.43	-4.97	9.11E-03
CG1667	CG1667	25.2	76.37	-2.07	9.19E-03
CG7966	CG7966	0.11	5.47	-4.92	9.20E-03
CG9455	CG9455	0	4.69	-5.52	9.29E-03
CG11217	CanB2	9.51	33.61	-2.28	9.31E-03
CG11086	Gadd45	4.14	18.58	-2.60	9.41E-03
CG32149	RhoGAP71E	10.57	36.29	-2.24	9.61E-03
CG31013	CG31013	0.77	8.16	-3.65	9.63E-03
CG15671	cv-2	11.75	39.48	-2.21	9.63E-03
CG12393	CG12393	0	4.62	-5.50	9.74E-03
CG1442	eIF4E-6	7.56	27.95	-2.34	9.78E-03
CG5337	CG5337	0	4.61	-5.49	9.80E-03
CG5123	W	1.07	9.13	-3.40	9.96E-03
CG42259	CG42259	0	4.58	-5.49	1.00E-02
CG13109	tai	12.01	39.82	-2.19	1.01E-02
CG9432	l(2)01289	0.86	8.37	-3.55	1.01E-02
CG32088	CG32088	11.49	38.35	-2.20	1.02E-02
CG6014	CG6014	117.1	317.77	-1.92	1.02E-02
CG32313	CG32313	0	4.54	-5.47	1.03E-02
CG5195	CG5195	0.02	4.67	-5.33	1.07E-02
CG11206	CG11206	0.05	4.88	-5.15	1.07E-02
CG13000	CG13000	12.16	39.66	-2.17	1.08E-02
CG1372	yl	0.01	4.55	-5.38	1.10E-02
CG8063	yellow-f2	0.2	5.64	-4.53	1.10E-02
CG40384	CG40384	0	4.44	-5.44	1.10E-02
CG10064	CG10064	0.13	5.27	-4.76	1.12E-02
CG13177	CG13177	0	4.42	-5.44	1.12E-02
CG3299	Vinc	7.21	26.25	-2.32	1.12E-02
CG41257	CG41257	0.37	6.34	-4.12	1.13E-02
CG7576	Rab3	0	4.4	-5.43	1.13E-02
CG1721	Pglym78	6.71	24.81	-2.34	1.14E-02
CG13047	CG13047	0	4.38	-5.42	1.15E-02
CG5287	CG5287	2	11.7	-2.94	1.15E-02
CG10116	CG10116	0.15	5.32	-4.68	1.15E-02
CG10931	CG10931	0	4.37	-5.42	1.16E-02
CG5387	Cdk5alpha	0.17	5.39	-4.60	1.17E-02
CG7208	CG7208	0	4.34	-5.41	1.18E-02
CG32221	CG32221	0	4.33	-5.41	1.19E-02
CG9595	osm-6	0.31	5.96	-4.21	1.21E-02
CG33099	CG33099	0.42	6.39	-4.00	1.22E-02
CG15326	CG15326	0	4.28	-5.39	1.23E-02
CG33178	CG33178	0	4.28	-5.39	1.23E-02
CG31246	CG31246	0	4.27	-5.39	1.24E-02
CG15261	UK114	2.86	13.96	-2.70	1.24E-02
CG3903	Gli	3.2	14.85	-2.64	1.26E-02
CG2759	w	5.9	22.13	-2.36	1.26E-02
CG1389	tor	0	4.24	-5.38	1.27E-02
CG2948	rev7	5.76	21.72	-2.36	1.28E-02
CG12951	CG12951	0	4.22	-5.37	1.28E-02
CG5272	gnu	0.06	4.66	-5.01	1.29E-02
CG32541	CG32541	12.94	40.22	-2.10	1.30E-02
CG12224	CG12224	2.76	13.52	-2.71	1.30E-02
CG42288	CG42288	0.16	5.17	-4.59	1.31E-02
CG33983	obst-H	0	4.19	-5.36	1.31E-02
CG9875	CG9875	0.27	5.66	-4.27	1.31E-02
CG8742	Gyc76C	0.57	6.82	-3.75	1.32E-02
CG32975	nAcRalpha-34E	0	4.17	-5.35	1.33E-02
CG10939	Sip1	22.5	64.21	-1.98	1.33E-02
CG4475	Idgf2	11.07	35.13	-2.13	1.35E-02
CG2977	inx7	0	4.14	-5.34	1.36E-02
CG17336	Lcch3	0	4.13	-5.34	1.37E-02
CG34171	CG34171	0	4.12	-5.34	1.38E-02
CG1505	gd	0	4.12	-5.34	1.38E-02
CG3401	betaTub60D	31.18	85.42	-1.93	1.39E-02
CG33106	mask	9.19	30.14	-2.17	1.39E-02
CG34120	CG34120	0.41	6.11	-3.96	1.40E-02
CG14290	CG14290	0	4.06	-5.32	1.44E-02
CG7886	CG7886	7.29	25.02	-2.23	1.46E-02
CG9331	CG9331	6.87	23.93	-2.25	1.46E-02
CG15678	CG15678	2.62	12.7	-2.69	1.50E-02
CG31299	nocturnin	3.03	13.73	-2.60	1.54E-02
CG18140	Cht3	0	3.96	-5.28	1.54E-02
CG32264	CG32264	9.45	30.1	-2.13	1.55E-02
CG11527	Tig	0.12	4.71	-4.66	1.55E-02
CG31017	PH4alphaNE3	0.07	4.44	-4.88	1.56E-02
CG1131	alpha-Est10	1.66	9.9	-2.95	1.56E-02
CG31370	CG31370	2.06	11.03	-2.81	1.56E-02
CG6775	rg	0.07	4.43	-4.87	1.57E-02
CG5779	Bc	0.02	4.11	-5.15	1.57E-02
CG10132	CG10132	0	3.91	-5.26	1.60E-02
CG14505	CG14505	5.54	20.11	-2.31	1.60E-02
CG6865	CG6865	0	3.9	-5.26	1.61E-02
CG17888	Pdp1	5.13	19.02	-2.33	1.62E-02
CG10184	CG10184	1.62	9.67	-2.95	1.63E-02
CG5501	Myo95E	0	3.88	-5.25	1.64E-02
CG32165	CG32165	0.77	7.07	-3.45	1.65E-02
CG31248	CG31248	1.23	8.47	-3.12	1.68E-02
CG9473	MED6	0	3.84	-5.24	1.68E-02
CG42389	CG42389	8.06	26.12	-2.15	1.69E-02
CG4970	CG4970	0	3.83	-5.23	1.70E-02
CG5004	CG5004	23.15	62.66	-1.91	1.70E-02
CG9640	CG9640	0	3.82	-5.23	1.71E-02
CG6986	CG6986	0.02	3.98	-5.10	1.73E-02
CG34411	CG34411	1.43	8.99	-3.01	1.73E-02
CG18408	CAP	8.1	26.06	-2.14	1.74E-02
CG1532	CG1532	5.53	19.7	-2.28	1.75E-02
CG30115	CG30115	0.46	5.87	-3.78	1.79E-02
CG3400	Pfrx	0	3.75	-5.20	1.80E-02
CG12449	Gfat1	1.3	8.5	-3.05	1.81E-02
CG12680	CG12680	0.64	6.45	-3.54	1.83E-02
CG11638	CG11638	0.15	4.6	-4.47	1.84E-02
CG34116	CG34116	0	3.72	-5.19	1.84E-02
CG17839	CG17839	0.18	4.72	-4.37	1.85E-02
CG18111	Obp99a	0.13	4.49	-4.54	1.86E-02
CG12950	CG12950	0.16	4.62	-4.43	1.87E-02
CG18547	CG18547	6.57	21.95	-2.19	1.87E-02
CG5304	l(2)01810	16.4	45.31	-1.93	1.91E-02
CG31437	CG31437	0	3.67	-5.17	1.91E-02
CG3616	Cyp9c1	3.82	15.03	-2.41	1.92E-02
CG14406	CG14406	0	3.65	-5.17	1.94E-02
CG30497	CG30497	4.3	16.18	-2.35	1.95E-02
CG42368	CG42368	0.29	5.09	-4.05	1.96E-02
CG12398	CG12398	0.12	4.36	-4.55	1.96E-02
CG14303	Qin	4.31	16.15	-2.34	1.98E-02
CG2706	fs(1)Yb	6.64	21.79	-2.17	2.00E-02
CG3502	CG3502	0.08	4.12	-4.70	2.02E-02
CG15267	CG15267	0	3.59	-5.14	2.02E-02
CG33197	mbl	17.66	47.62	-1.90	2.04E-02
CG11066	scarface	5.15	18.01	-2.25	2.08E-02
CG31999	CG31999	2.56	11.54	-2.58	2.11E-02
CG10026	CG10026	4.42	16.17	-2.31	2.11E-02
CG7851	Scgalpha	2.95	12.51	-2.50	2.12E-02
CG15329	hdm	0.18	4.51	-4.30	2.13E-02
CG1098	Madm	1.83	9.59	-2.77	2.14E-02
CG10594	spo	2.54	11.44	-2.58	2.14E-02
CG13005	CG13005	0	3.51	-5.11	2.15E-02
CG14865	l(3)neo43	0	3.49	-5.10	2.18E-02
CG10537	Rdl	0.02	3.65	-4.98	2.19E-02
CG31344	CG31344	0	3.48	-5.10	2.20E-02
CG3066	Sp7	2.44	11.1	-2.59	2.21E-02
CG31973	CG31973	0	3.46	-5.09	2.23E-02
CG9821	CG9821	17.93	47.3	-1.87	2.24E-02
CG15330	CG15330	1.39	8.28	-2.93	2.24E-02
CG33100	4EHP	7.14	22.35	-2.10	2.27E-02
CG10573	ko	0	3.44	-5.08	2.27E-02
CG32181	CG32181	0	3.42	-5.08	2.30E-02
CG9761	Nep2	0.17	4.35	-4.30	2.30E-02
CG30429	CG30429	0	3.4	-5.07	2.34E-02
CG9505	CG9505	1.81	9.29	-2.74	2.36E-02
CG41254	CG41254	0	3.38	-5.06	2.37E-02
CG32549	CG32549	11.79	32.78	-1.94	2.37E-02
CG5550	CG5550	0.62	5.9	-3.45	2.39E-02
CG30413	CG30413	0	3.37	-5.05	2.39E-02
CG4190	Hsp67Bc	0.83	6.52	-3.24	2.42E-02
CG11897	CG11897	23.78	59.5	-1.79	2.45E-02
CG31093	CG31093	0	3.33	-5.04	2.46E-02
CG10946	dpr14	0.2	4.37	-4.17	2.47E-02
CG15848	Scp1	5.96	19.17	-2.14	2.48E-02
CG2184	Mlc2	0.38	5.01	-3.76	2.53E-02
CG42255	CG42255	1.15	7.35	-3.00	2.53E-02
CG4965	twe	0	3.26	-5.01	2.60E-02
CG6642	a10	0	3.26	-5.01	2.60E-02
CG11238	l(3)04053	1.31	7.73	-2.90	2.60E-02
CG14622	DAAM	0	3.25	-5.00	2.62E-02
CG4068	CG4068	18.24	46.34	-1.81	2.64E-02
CG1322	zfh1	13.98	36.89	-1.87	2.64E-02
CG4669	CG4669	0.05	3.58	-4.71	2.65E-02
CG17207	CG17207	0.64	5.77	-3.38	2.66E-02
CG11019	CG11019	0.45	5.16	-3.62	2.67E-02
CG13780	Pvf2	5.46	17.7	-2.14	2.67E-02
CG32040	CG32040	0	3.22	-4.99	2.68E-02
CG9592	CG9592	0	3.21	-4.99	2.70E-02
CG9572	CG9572	0	3.21	-4.99	2.70E-02
CG10621	CG10621	0.15	4.03	-4.28	2.70E-02
CG33144	CG33144	4.14	14.58	-2.25	2.71E-02
CG4607	CG4607	0.03	3.43	-4.81	2.72E-02
CG32695	CG32695	0	3.2	-4.98	2.72E-02
CG1522	cac	9.12	25.85	-1.96	2.73E-02
CG34360	CG34360	1.04	6.87	-3.03	2.76E-02
CG11607	H2.0	0	3.18	-4.97	2.77E-02
CG31436	CG31436	0.92	6.52	-3.11	2.77E-02
CG4827	CG4827	0.48	5.19	-3.56	2.78E-02
CG18525	Spn5	61.47	139.73	-1.66	2.79E-02
CG6999	CG6999	4.15	14.44	-2.24	2.84E-02
CG11448	CG11448	5.52	17.53	-2.11	2.87E-02
CG2904	ec	4.41	15	-2.21	2.87E-02
CG6383	crb	1.06	6.83	-3.00	2.89E-02
CG12592	CG12592	0	3.11	-4.94	2.92E-02
CG5888	CG5888	15.59	39.51	-1.81	2.94E-02
CG15422	CG15422	0	3.1	-4.94	2.94E-02
CG8229	CG8229	7.65	22.15	-1.99	2.95E-02
CG8308	alphaTub67C	0.4	4.82	-3.65	2.96E-02
CG6847	CG6847	0.33	4.57	-3.77	2.99E-02
CG1483	Map205	29.32	68.82	-1.70	3.00E-02
CG4385	S	3.35	12.39	-2.31	3.01E-02
CG8938	GstS1	34.85	80.57	-1.68	3.02E-02
CG6643	CG6643	0.35	4.62	-3.73	3.02E-02
CG14478	CG14478	4.15	14.21	-2.21	3.03E-02
CG11505	CG11505	19.98	48.64	-1.75	3.04E-02
CG4760	bol	0.73	5.79	-3.23	3.06E-02
CG4622	CG4622	0	3.05	-4.92	3.06E-02
CG8667	dimm	1.17	7.01	-2.91	3.06E-02
CG3359	mfas	10.97	29.18	-1.87	3.07E-02
CG31693	CG31693	0.24	4.2	-3.96	3.08E-02
CG14856	CG14856	0	3.04	-4.91	3.09E-02
CG15561	CG15561	0	3.04	-4.91	3.09E-02
CG3409	CG3409	7.41	21.38	-1.98	3.09E-02
CG8421	Asph	1.2	7.05	-2.89	3.12E-02
CG1623	CG1623	0.13	3.74	-4.28	3.12E-02
CG3916	CG3916	0.09	3.56	-4.44	3.12E-02
CG31691	TotF	0	3.02	-4.90	3.13E-02
CG14745	PGRP-SC2	0.17	3.9	-4.14	3.14E-02
CG8920	CG8920	21.34	51.13	-1.73	3.16E-02
CG32529	CG32529	5.9	17.93	-2.05	3.18E-02
CG34341	Pde11	19.99	48.13	-1.74	3.20E-02
CG13035	CG13035	1.68	8.21	-2.66	3.22E-02
CG4579	Nup154	10.63	28.08	-1.86	3.24E-02
CG41451	CG41451	0	2.97	-4.88	3.26E-02
CG12460	CG12460	0	2.96	-4.87	3.29E-02
CG33252	CG33252	0	2.96	-4.87	3.29E-02
CG8340	128up	12.64	32.24	-1.82	3.30E-02
CG11895	stan	0.6	5.27	-3.33	3.30E-02
CG4120	Cyp12c1	0.96	6.29	-3.01	3.31E-02
CG42346	CG42346	0.09	3.48	-4.40	3.31E-02
CG2668	Peb	0.11	3.57	-4.32	3.32E-02
CG32743	Smg1	2.7	10.57	-2.38	3.33E-02
CG8994	exu	0	2.94	-4.86	3.34E-02
CG13826	CG13826	2.16	9.29	-2.50	3.34E-02
CG30288	CG30288	0	2.93	-4.86	3.37E-02
CG7850	puc	8.97	24.29	-1.90	3.37E-02
CG42345	CG42345	2.08	9.07	-2.52	3.38E-02
CG11617	CG11617	0	2.92	-4.85	3.39E-02
CG9559	fog	0.03	3.13	-4.68	3.42E-02
CG7100	CadN	6.62	19.18	-1.99	3.42E-02
CG8714	sut1	2.93	11.03	-2.33	3.42E-02
CG7892	nmo	21.37	50.3	-1.70	3.42E-02
CG5375	CG5375	3.55	12.42	-2.24	3.44E-02
CG7322	CG7322	0	2.9	-4.85	3.45E-02
CG31811	cenG1A	25.71	59.2	-1.67	3.46E-02
CG7029	CG7029	4.92	15.42	-2.09	3.47E-02
CG9411	CG9411	5.02	15.63	-2.08	3.48E-02
CG16936	CG16936	41.8	92.44	-1.62	3.50E-02
CG8102	CG8102	0.55	5.01	-3.36	3.53E-02
CG5144	CG5144	0.36	4.41	-3.64	3.54E-02
CG34034	CG34034	2.08	8.95	-2.50	3.55E-02
CG3315	TrxT	0.05	3.18	-4.55	3.58E-02
CG5285	CG5285	0	2.85	-4.82	3.59E-02
CG5657	Scgbeta	0.09	3.37	-4.36	3.60E-02
CG33984	CG33984	0	2.84	-4.82	3.62E-02
CG7693	fray	10	26.04	-1.84	3.63E-02
CG17264	CG17264	3.22	11.51	-2.26	3.63E-02
CG1765	EcR	10.83	27.75	-1.82	3.64E-02
CG9262	Shal	0.47	4.71	-3.44	3.65E-02
CG6930	l(3)neo38	6.45	18.47	-1.97	3.68E-02
CG32172	noe	10.53	27.04	-1.82	3.69E-02
CG32580	CG32580	0.01	2.9	-4.75	3.70E-02
CG13194	pyr	8.84	23.48	-1.87	3.71E-02
CG7320	CG7320	0.03	3.02	-4.63	3.72E-02
CG16899	CG16899	0.03	3.02	-4.63	3.72E-02
CG1469	Fer2LCH	75.72	160.4	-1.56	3.73E-02
CG1268	CG1268	0	2.8	-4.80	3.74E-02
CG18173	CG18173	0.14	3.53	-4.15	3.75E-02
CG4551	smi35A	7.4	20.39	-1.92	3.75E-02
CG18584	koi	0.85	5.75	-3.03	3.76E-02
CG11664	CG11664	0	2.79	-4.79	3.77E-02
CG32573	CG32573	0	2.79	-4.79	3.77E-02
CG10006	CG10006	0	2.78	-4.79	3.80E-02
CG9427	CG9427	0.17	3.63	-4.04	3.80E-02
CG32185	CG32185	3.42	11.77	-2.21	3.85E-02
CG33936	CG33936	51.66	110.5	-1.57	3.85E-02
CG5194	CG5194	0.17	3.61	-4.03	3.86E-02
CG13771	CG13771	0.27	3.97	-3.77	3.87E-02
CG11275	CG11275	0.22	3.79	-3.89	3.88E-02
CG32772	CG32772	7.72	20.89	-1.89	3.88E-02
CG12265	Det	9.62	24.82	-1.83	3.89E-02
CG7734	shn	8.41	22.31	-1.87	3.89E-02
CG42241	CG42241	0	2.74	-4.77	3.92E-02
CG8817	lilli	3.78	12.49	-2.16	3.93E-02
CG13516	CG13516	0.39	4.34	-3.53	3.94E-02
CG5661	Sema-5c	20.01	45.8	-1.66	4.00E-02
CG10593	Acer	4.32	13.59	-2.09	4.01E-02
CG34460	CG34460	0	2.71	-4.75	4.02E-02
CG32319	CG32319	0	2.71	-4.75	4.02E-02
CG11658	CG11658	3.67	12.17	-2.16	4.03E-02
CG40409	CG40409	1.47	7.19	-2.65	4.03E-02
CG16716	CG16716	0	2.7	-4.75	4.05E-02
CG1385	Def	0	2.69	-4.74	4.09E-02
CG5431	CG5431	8.49	22.21	-1.85	4.09E-02
CG17803	CG17803	1.65	7.59	-2.58	4.09E-02
CG17608	fu12	0.92	5.78	-2.94	4.09E-02
CG8366	CG8366	2.62	9.8	-2.32	4.12E-02
CG17530	GstE6	105.89	217.2	-1.51	4.12E-02
CG30106	CG30106	2.6	9.75	-2.32	4.13E-02
CG6737	CG6737	0.15	3.43	-4.06	4.15E-02
CG12851	CG12851	0.01	2.75	-4.68	4.17E-02
CG31057	tau	0	2.66	-4.72	4.19E-02
CG40091	CG40091	0	2.66	-4.72	4.19E-02
CG4596	CG4596	0.1	3.21	-4.23	4.20E-02
CG12199	kek5	4.35	13.48	-2.07	4.21E-02
CG14879	CG14879	2.16	8.7	-2.41	4.22E-02
CG11466	Cyp9f2	4.73	14.27	-2.04	4.22E-02
CG34220	CG34220	0	2.65	-4.72	4.22E-02
CG3698	CG3698	0	2.65	-4.72	4.22E-02
CG12342	dgo	1.38	6.87	-2.67	4.23E-02
CG17129	CG17129	3.26	11.11	-2.20	4.26E-02
CG10280	CG10280	1.47	7.06	-2.63	4.29E-02
CG3166	aop	4.5	13.73	-2.05	4.29E-02
CG30485	CG30485	0	2.63	-4.71	4.29E-02
CG5151	CG5151	3.29	11.12	-2.18	4.34E-02
CG7841	CG7841	9.68	24.3	-1.79	4.34E-02
CG7951	sima	15.58	36.08	-1.68	4.36E-02
CG17131	SP71	1.56	7.23	-2.58	4.37E-02
CG10174	Ntf-2r	0	2.6	-4.69	4.40E-02
CG13598	sba	1.96	8.14	-2.45	4.41E-02
CG42469	Sfp26Ac	0	2.59	-4.69	4.44E-02
CG7452	Syx17	0	2.59	-4.69	4.44E-02
CG8646	CG8646	0.28	3.8	-3.67	4.45E-02
CG34359	IP3K2	28.47	61.35	-1.58	4.46E-02
CG17077	pnt	1.99	8.17	-2.43	4.48E-02
CG4324	CG4324	1.19	6.29	-2.74	4.48E-02
CG31349	pyd	39.64	83.21	-1.54	4.49E-02
CG8600	CG8600	5.84	16.31	-1.93	4.51E-02
CG11866	CG11866	0.42	4.22	-3.41	4.52E-02
CG10868	orb	0.16	3.34	-3.97	4.57E-02
CG31772	CG31772	0.03	2.76	-4.51	4.58E-02
CG33698	CG33698	0	2.55	-4.67	4.59E-02
CG10763	Gbeta5	3.53	11.45	-2.13	4.60E-02
CG3655	CG3655	5.67	15.87	-1.93	4.61E-02
CG30217	CG30217	0	2.53	-4.66	4.67E-02
CG2082	CG2082	0	2.53	-4.66	4.67E-02
CG42379	CG42379	5.42	15.29	-1.94	4.70E-02
CG8588	pst	23.56	50.99	-1.58	4.72E-02
CG32062	CG32062	13.97	32.24	-1.67	4.73E-02
CG11501	CG11501	0	2.51	-4.64	4.75E-02
CG7847	sr	0.05	2.82	-4.38	4.75E-02
CG32373	CG32373	0.03	2.71	-4.48	4.77E-02
CG13565	CG13565	0	2.5	-4.64	4.79E-02
CG33555	btsz	0	2.5	-4.64	4.79E-02
CG4783	CG4783	0	2.5	-4.64	4.79E-02
CG14982	CG14982	4.1	12.5	-2.05	4.80E-02
CG10269	D19A	3.71	11.67	-2.09	4.83E-02
CG9381	mura	3.89	12.03	-2.06	4.85E-02
CG34144	CG34144	0.2	3.39	-3.82	4.90E-02
CG5707	CG5707	2.41	8.86	-2.29	4.93E-02
CG1146	CG1146	4.57	13.37	-1.99	4.93E-02
CG9755	pum	12.28	28.62	-1.69	4.94E-02
CG1981	Thd1	21.89	47.23	-1.58	4.94E-02
CG1102	MP1	0	2.46	-4.62	4.95E-02
CG10118	ple	4.2	12.58	-2.02	4.98E-02
CG9778	CG9778	0.36	3.89	-3.46	4.99E-02
CG5585	CG5585	0	2.45	-4.61	4.99E-02

Supplemental Table S3. List of siRNAs and primers. RNA is shown in italics.

experiment	Name	sense	antisense
RNAi in OSC and Δmbt-OSC	siEGFP	*GGCAAGCUGACCCUGAAGU*TT	*ACUUCAGGGUCAGCUUGCC*TT
	siL(3)mbt-1	*CCUCUGGAGAUGUCCGUAA*TT	*UUACGGACAUCUCCAGAGG*TT
	siL(3)mbt-2	*CCACCGGUGGUCAAGGAAA*TT	*UUUCCUUGACCACCGGUGG*TT
	siLuc	*CGUACGCGGAAUACUUCGA*TT	*UCGAAGUAUUCCGCGUACG*TT
	siVasa	*GCAGACCAUAUACGAAGUU*TT	*AACUUCGUAUAUGGUCUGC*TT

experiment	Name	Primer sequence
RNAi in S2	dsRNA against *l(3)mbt*	TAATACGACTCACTATAGGGAGAGGTTCGAGCTGAATGAG
		TAATACGACTCACTATAGGGAGAGATCTCGAAGCAGTTGG

experiment	Name	forward primer (5' > 3')	reverse primer (5' > 3')
qRT-PCR	*vasa*	AGCCGTCCAACGATGCA	AACATCGCTGCCGGTCA
	ago3	CTGCATTTGTGGCCTCCATA	GGGGAGTTTGCCATTCCTTT
	piwi	CAAGGCCGGATAATTGGACA	CCATCGCTCGGAGTGGTAAG
	l(3)mbt	GCCCCCGAGAATATCAGACG	AGGAGACGTCTTTCGCTTGG

experiment	Name	forward primer (5' > 3')	reverse primer (5' > 3')
Genomic PCR	*l(3)mbt*	TACGACTTCTGGGTGAACGC	GACATCCCTCCACATCGCAA

experiment	Name	Primer sequence	plasmid
plasmid construction	VasRA-KpnI-F	TTTTGGTACCATGTCTGACGACTGGGATGATG	pAcM-Vasa
	VasRA-XhoI-R	TTTTCTCGAGATCCCATTGCTCTTCTTCCTC	pAcM-Vasa
	CRISPR-l3mbt-E2-F1	CAGCTGCACCGTTTTAGAGCTAGAAATAGC	sgRNA E2
	CRISPR-l3mbt-E2-R2	AACCCGGTAACAGTATTGAGG	sgRNA E2
	CRISPR-l3mbt-GM76-F1	AGCCGCTATTGTTTTAGAGCTAGAAATAGC	sgRNA GM76
	CRISPR-l3mbt-GM76-R2	CCAACTGAAGCAGTATTGAGG	sgRNA GM76
